# Identification of specific transcripts for plant heat stress tolerance through genome-wide analysis of SIZ1-dependent alternative polyadenylation

**DOI:** 10.1007/s44154-026-00340-2

**Published:** 2026-08-18

**Authors:** Jun Wang, Xiujuan Wu, Zhou Zhou, Sitong Zheng, Minghui Hu, Yincui Xiao, Leqian Shi, Cheng Zhang, Jiamei Li, Chengwei Yang, Jianbin Lai, Danlu Han, Zhibo Yu

**Affiliations:** Guangdong Provincial Key Laboratory of Biotechnology for Plant Development, School of Life Science, South China Normal University, Guangzhou, 510631 China

**Keywords:** Heat stress, Alternative polyadenylation, *SIZ1*, *GolS2*, *TTL3*

## Abstract

Heat stress (HS) has emerged as a significant environmental factor affecting plant growth and agricultural productivity. Alternative polyadenylation (APA) is a crucial co-transcriptional process that regulates developmental processes and stress responses in plants. However, the distinct roles of its resultant transcripts in plant HS response remain to be investigated. In this study, we employed poly(A) tag sequencing (PAT-seq) to identify over 1500 transcripts whose expression exhibited significant alterations in response to HS, mediated by SIZ1, a SUMO E3 ligase in Arabidopsis. Further analysis revealed that more than 300 switch genes with varying poly(A) site usages were differentially expressed under HS conditions. Compared to non-canonical poly(A) sites, more genes utilize 3’UTR sites to regulate transcript expression by altering the usage of poly(A) signals. Based on the information of APA genes regulated by SIZ1, we selected two genes, *Galactinol synthase enzyme* (*GolS2*) and *Tetratricopeptide repeat (TPR) like 3* (*TTL3*), for characterizing their novel functions. Overexpression of the distal transcript of *GolS2* or the proximal transcript of *TTL3* enhanced heat tolerance in Arabidopsis. Collectively, our current study elucidates the regulation of APA mediated by SIZ1 during HS response and establishes a strategy for identifying specific transcripts arising from APA for plant heat tolerance.

**Supplementary Information:**

The online version contains supplementary material available at 10.1007/s44154-026-00340-2.

## Introduction

Global climate change with rising temperatures has great impacts on plant growth and yield production (Lesk et al. 2016; Lobell et al. 2011). In response to various high-temperature conditions, plants undergo a range of processes, including thermomorphogenesis in warm ambient temperatures and heat stress (HS) responses under extreme high temperatures (Hayes et al. 2021; Li et al. 2019). Under HS conditions, plants suffer severe injuries due to the loss of membrane integrity, inactivation of proteins, production of reactive oxygen species, and disruption of metabolic pathways (Fragkostefanakis et al. 2015; Zhang et al. 2024). Therefore, an efficient signaling transduction network has evolved in plants to enhance their survival under HS (Ding et al. 2020). In the core of this network, essential transcription factors meticulously regulate the transcription of HS-inducible genes. Given that in plant cells, pre-mRNAs undergo a series of processing steps, including 5’ capping, splicing, and 3’ polyadenylation, the functions of different mature mRNAs may vary and it is crucial to identify specific transcript isoforms for plant HS tolerance.

The polyadenylation (poly(A)) of the 3’ end of pre-mRNA represents a crucial co-transcriptional processing step in the eukaryotic mRNA maturation (Deng and Cao 2017; Mitschka and Mayr 2022). Moreover, over 50% of eukaryotic genes possess multiple poly(A) sites within the same gene, a phenomenon referred to as alternative polyadenylation (APA) and is very pervasive in eukaryotes (Fu et al. 2016; Hoque et al. 2013; Ozsolak et al. 2010; Wu et al. 2011). APA is primarily regulated by the polyadenylation factor complex and the signal cis-elements situated upstream or near the poly(A) site (Hunt et al. 2008; Loke et al. 2005). In mammals, 50% of genes use the dominant poly(A) signal, the hexamer “AAUAAA” (Chan et al. 2014). However, the poly(A) signal “AAUAAA” only occupies about 10% of the Near Up Element (NUE) region, which is located between −35 to −10 bp upstream of poly(A) sites, and its one-nucleotide variants only about 30% in plants (Hao et al. 2023).

APA contributes to the diversity and complexity of transcriptomes, playing a crucial role in regulating the expression of eukaryotic genes (Xing and Li 2011). APA achieves this by regulating the coding sequence (CDS) and the length of the 3’ untranslated region (3’UTR). When APA occurs in the upstream regions of mRNA, it often results in the production of a truncated protein, which can impair the function of the full-length protein. If APA occurs in the 3’UTR, it may alter mRNA stability or translation efficiency (Elkon et al. 2013). APA events are linked to developmental processes and stress responses across various plant species (Hunt 2020; Wu et al. 2023; You et al. 2021; Zhou and Li 2023). Recently, we reported on the role of SUMOylation in regulating APA that is involved in plant thermomorphogenesis under warm ambient temperature conditions (Yu et al. 2024). However, specific transcripts with distinct poly(A) sites involved in HS response, as revealed by mRNA 3’ end PAT-seq transcriptome analysis, remain to be identified in Arabidopsis.

SUMOylation is an important post-translational modification that covalently attaches small ubiquitin-like modifiers (SUMOs) to lysine residues of substrates via an E1-E2-E3 enzyme cascade (Miura et al. 2007; Park et al. 2011). The SUMO E3 ligase, SIZ1, plays a pivotal role in regulating development and stress responses in plants (Augustine and Vierstra 2018; Niu et al. 2019). During HS, SIZ1 mediates the regulation of chromatin-associated SUMO signals at promoter-transcriptional start site regions for the expression of heat-responsive genes (Han et al. 2021). Furthermore, NF-YC10 functions as a key regulator in plant HS response, and SIZ1 enhances the SUMOylation of NF-YC10 to modulate transcription for HS tolerance in Arabidopsis (Huang et al. 2023; Li et al. 2025). These results suggest that SIZ1 is involved in transcriptional regulation during HS, but it remains unclear whether SIZ1 plays a crucial role in co-transcriptional polyadenylation in this process. Recently, we reported that SIZ1 regulates the SUMOylation of CPSF100, which modulates APA and is involved in plant’s responses to warm ambient temperature conditions (Yu et al. 2024). If SIZ1 also contributes to APA under HS, based on the role of this SUMO ligase in heat tolerance, it would provide valuable insights into identifying specific transcripts associated with HS responses.

Here, we found that HS remodels APA patterns to drive the production of specific transcript isoforms in Arabidopsis. Interestingly, this regulatory mechanism is mediated by SIZ1. Based on the genome-wide data analysis, we selected two candidate APA genes, *GolS2* and *TTL3*, whose proximal/distal expression was increased and decreased respectively in the *siz1-2* mutant plants, for further functional characterization. The phenotypic data indicated that the overexpression of the distal transcript of *GolS2* or the proximal transcript of *TTL3* enhanced heat tolerance. Therefore, our current work support that plants respond to HS through the switching of alternative proximal and distal poly(A) sites and provide a strategy for identification of specific transcripts for plant HS tolerance.

## Results

### Alternative polyadenylation mediated by SIZ1 was prevalent in response to heat stress

**Fig. 1:**
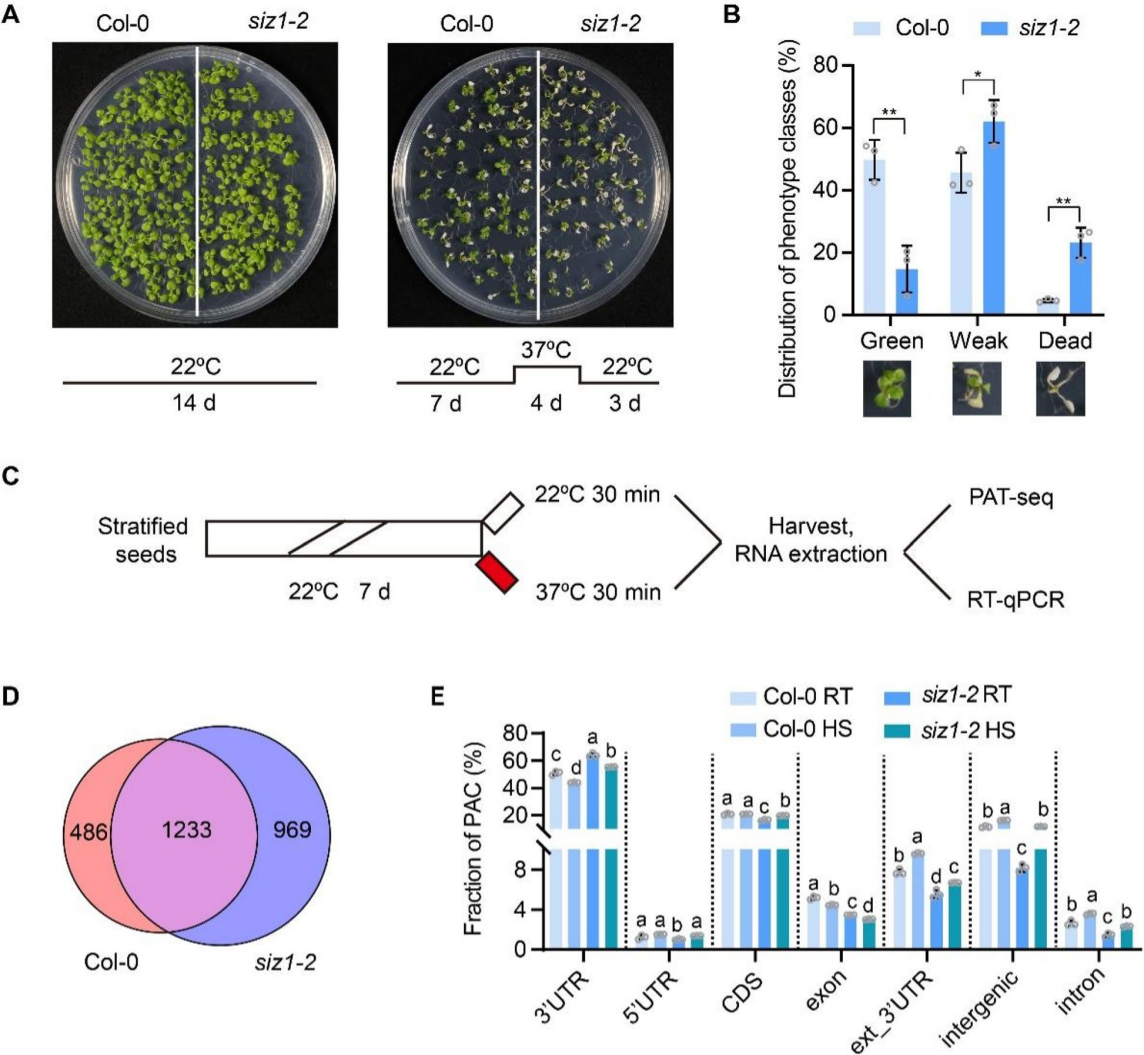
Analysis of SIZ1-dependent DE-PACs in responses to HS. **A-B** The phenotype of *siz1-2* under HS treatments. Seven-day-old wild-type (Col-0) and *siz1-2* seedlings were subjected to a treatment at 37 °C for 4 days in the incubator with 60% relative humidity, followed by a recovery period of 3 d at 22 °C before being photographed. The seedlings remained at 22 °C were used as the control group. The representative phenotypic changes observed following recovery are shown in **A**. The percentages of green, weak (characterized by only two green leaves), and dead seedlings are shown in **B**. Data are mean ± SD from three biologically independent experiments. Three biologically independent experiments showed similar patterns. Significant differences were analyzed using Student’s *t*-test (*p < 0.05; **p < 0.01). **C** The protocol scheme of the heat treatment used in this study. Total RNA extracted from seedlings was subject to PAT-seq for APA analysis and RT-qPCR for transcript expression assessment. **D** Overlap of DE-PACs between Col-0 and *siz1-2*. An adjusted *p* value < 0.05 was used to identify significant DE-PACs. **E** Distributions of DE-PACs at the genome level. Significant differences were analyzed using one-way ANOVA followed by Tukey’s multiple comparisons test (*p* < 0.05)

Given that previous studies have demonstrated the involvement of SIZ1 in heat tolerance in Arabidopsis, we aimed to establish a system for investigating the role of SIZ1 in APA in response to HS. To this end, we assessed the phenotype of the *siz1-2* mutant at 37 °C. Consistent with the previous report at 39 °C (Yoo et al. 2006), the proportion of green seedlings of the *siz1-2* mutant was significantly lower than that observed in the wild type. Additionally, there was a higher incidence of weak and dead seedlings in the mutant group (Fig. 1A, B). Therefore, conducting the treatment at 37 °C is appropriate for further analysis of SIZ1-dependent APA during heat stress responses. To elucidate the distinct transcript profiles that exhibit changes under HS, seedlings of the Col-0 and *siz1-2* mutant were subjected to treatments at 22 °C or 37 °C for a duration of 30 min prior to collection for PAT-seq analysis (Fig. 1C). A summary of the raw reads, mapping reads, poly(A) sites (poly(A) site clusters, PACs) and other relevant metrics for each library is provided in Data S1A. After raw data processing, 49,063 PACs were identified, as detailed in Data S1B. These PACs were mapped to 18,229 genes, with 67% exhibited more than one poly(A) site and were classified as APA genes. More than 60% of the PACs and over 40% of the PATs were located in the 3’UTRs (Fig. S1), which is similar to previous findings (Yu et al. 2019).

**Fig. 2:**
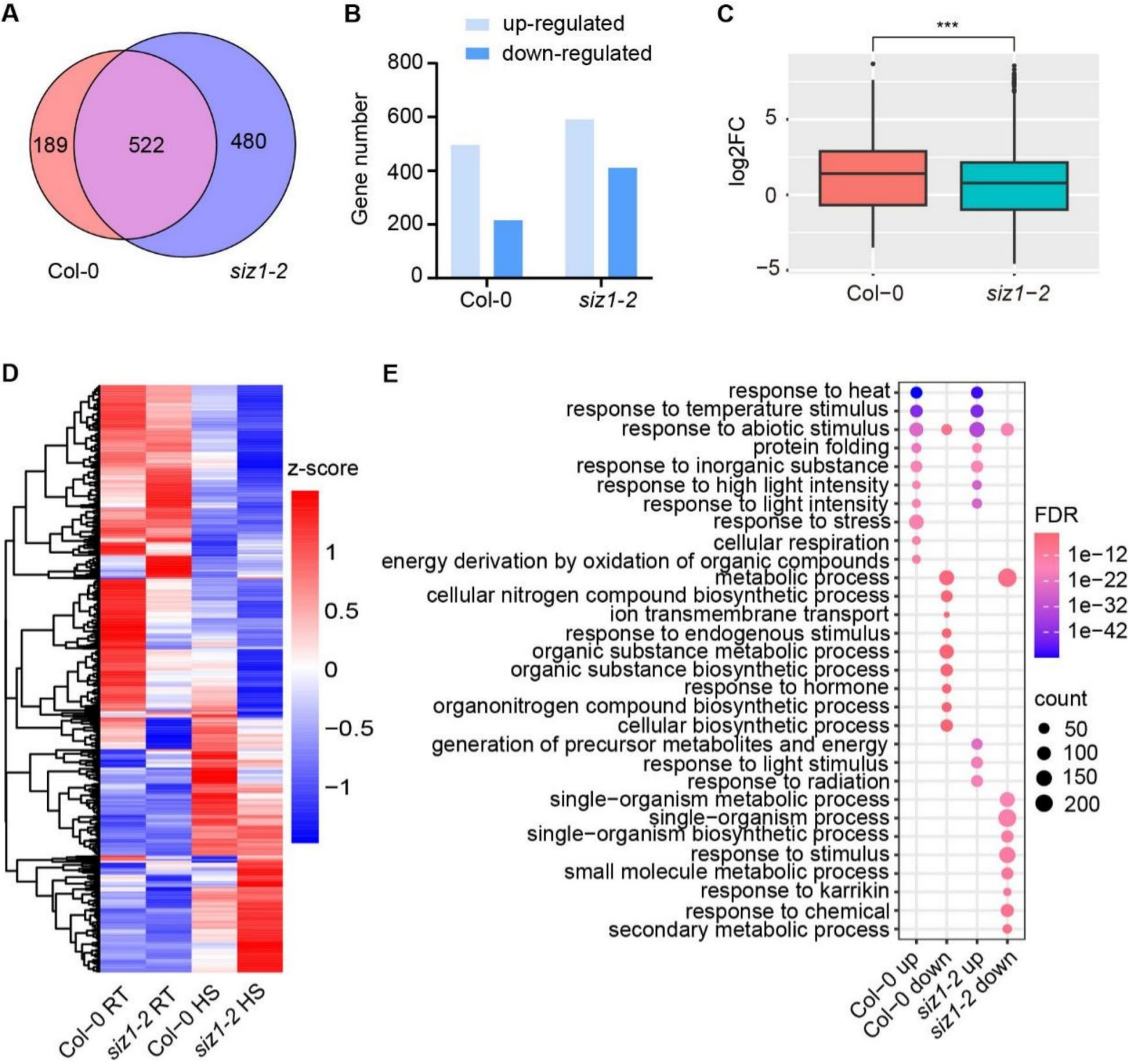
APA genes mediated by SIZ1 was widespread in response to HS. **A** Analysis of differentially expressed APA genes following heat treatments. DE-PAC genes with more than one poly(A) sites were defined as DE-APA genes. **B** The number of up-regulated and down-regulated DE-APA genes in Col-0 and *siz1-2* in response to HS. **C** A boxplot displaying the expression levels of DE-APA genes expression in HS response, where a log2 fold change (log2FC) < 0 indicates down-regulation, while > 0 indicates up-regulation. 189 + 522 genes in Col-0, 522 + 480 genes in *siz1-2.* Differential significance analysis was performed using the TukeyHSD function. *padj* value < 0.001 as shown ***. **D** A heatmap analysis depicting expression levels of DE-APA genes across samples. **E** GO analysis of up-regulated and down-regulated DE-APA genes in response to HS. 189 + 522 genes in Col-0, 522 + 480 genes in *siz1-2.* The top 10 significant GO terms related to biological processes are presented. A false discovery rate (FDR)-corrected *p* < 0.05 was considered statistically significant. The size of each dot represents the number of associated genes, while color indicates significance level

A total of 1719 differentially expressed (DE) PACs (hereafter referred to as DE-PACs, differentially expressed transcripts) were identified in Col-0 and 2202 in *siz1-2* following HS when compared to control samples (Data S1 C, D). Venn analysis showed that SIZ1 specifically regulated the expression of more than 900 transcripts (Fig. 1D). Furthermore, in comparison to room temperature (RT), HS resulted in an increase in the number of DE-PACs in ext_3’UTRs, intron, and intergenic regions, but there was a decrease observed in the numbers found in 3’UTR and exons (Fig. 1E). DE-PAC containing genes were termed as DE-PAC genes, and DE-PAC genes with multiple poly(A) sites were classified as DE-APA genes (Data S1 E, F). Differential expression analysis revealed that HS altered the expression of > 700 DE-APA genes, and 189 DE-APAs was SIZ1-dependent. However, the deficiency of *SIZ1* changed the expression of 480 DE-APA genes (Fig. 2A). These results suggested that SIZ1 regulates the expression of DE-APA genes under heat stress. Gene expression levels were quantified by summing the total counts of PATs associated with each gene (Data S1G), and the differentially expressed genes were defined DE genes. Furthermore, a substantial proportion of these DE genes overlapped with DE-APA genes (Fig. S2), supporting the importance of APA in regulating gene expression in response to HS. Interestingly, compared to down-regulated DE-APA gene in Col-0, more DE-APA genes were up-regulated during HS, and the ratio of up-regulated expression to down-regulated expression exceeds two times (488/221) (Fig. 2B). Although, the deletion of *SIZ1* altered expression of more DE-APA genes expression, the ratio was attenuated (591/411). Box plot and Heatmap analysis further demonstrated that HS significantly influenced the expression levels of DE-APA genes, while SIZ1 deficiency had further effects on these alterations (Fig. 2C, D). Subsequently, we investigated the potential functions of these DE-APA genes through gene ontology (GO) enrichment analyses and KEGG pathway analyses. The GO analysis indicated that the terms such as “response to heat” and “response to temperature stimulus” were significantly enriched among up-regulated DE-APA genes, supporting our findings that HS increases the expression levels of these DE-APA genes (Fig. 2E). Furthermore, distinct KEGG pathways were identified between in down-regulated and up-regulated DE-APA genes in Col-0, with “plant hormone signal transduction” was enriched in down-regulated DE-APA genes. Down-regulated DE-APA genes in *siz1-2* were significantly enriched in several specific KEGG pathways, including “biosynthesis of amino acids” and “porphyrin and chlorophyll metabolism” (Fig. S3). Collectively, these results indicate that genome-wide APA in response to HS is regulated by SIZ1.

### Heat stress widely influenced the utilization of poly(A) sites in the switch genes

**Fig. 3:**
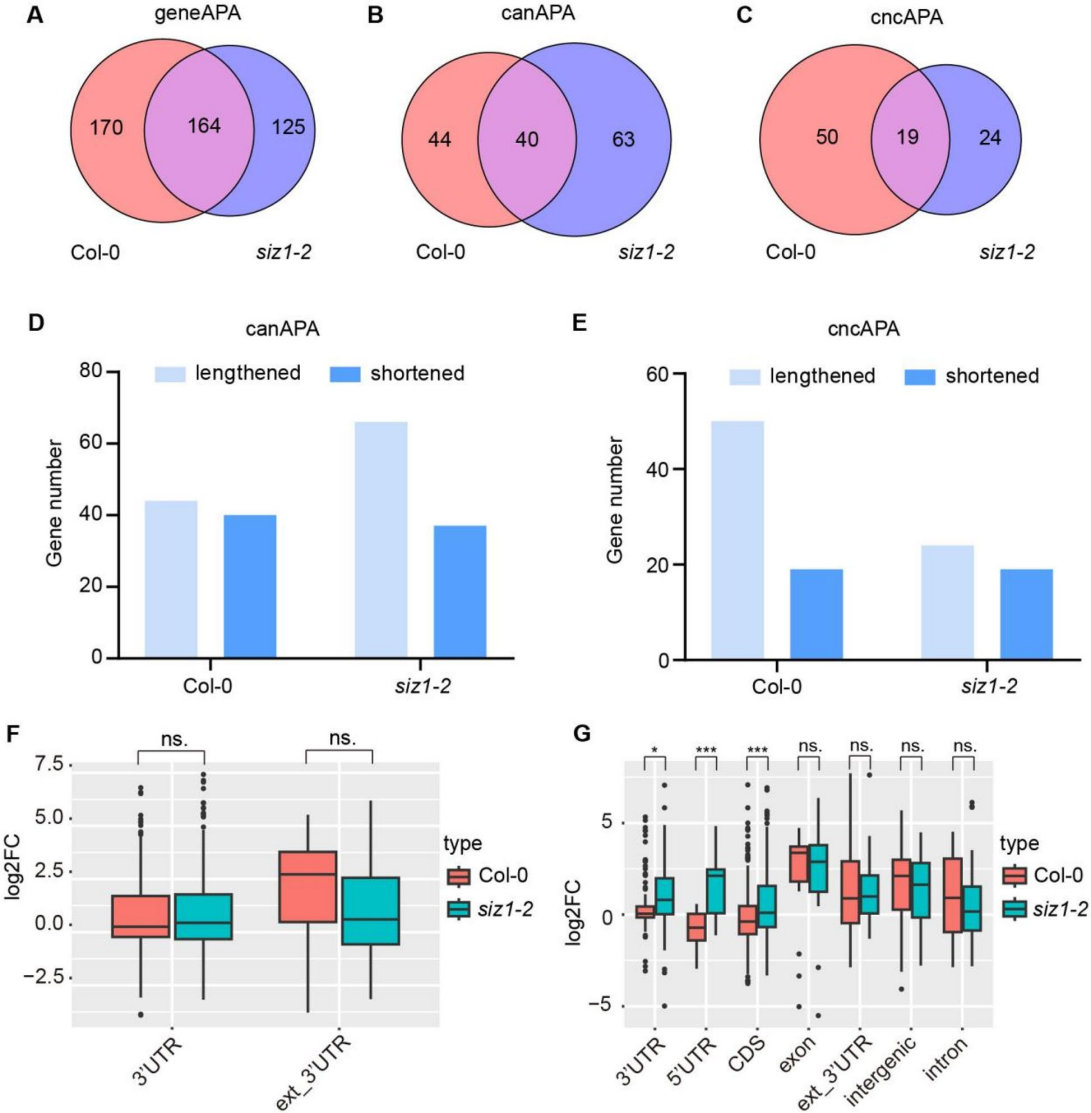
HS alters the poly(A) site usage of switch genes mediated by SIZ1. **A-C** The Venn diagrams which illustrate the response of switch genes to HS in both Col-0 and *siz1-2* backgrounds. A *p*-value of less than 0.05 was considered statistically significant for poly(A) sites usage of switch genes. GeneAPA quantifies APA dynamics among all PACs associated with a specific gene, treating with each PAC equally. CanAPA quantifies APA dynamics among canonical PACs located at the 3’UTR of a specific gene. CncAPA quantifies APA dynamics between two categories of PACs, including both canonical and non-canonical PACs in a gene. Non-canonical PACs refer to those located in intragenic regions excluding the 3’UTR. **D-E** The counts of shortened and lengthened switch genes as determined by with canAPA or cncAPA. **F** A boxplot diagram displaying the expression levels of transcripts located in genomic regions for switch genes with canAPA. Genes with annotated 3’UTRs were extended for 120 nucleotides, while genes without annotated 3’UTRs were extended by 338 nucleotides. A log2 fold change (log2FC) < 0 indicates down-regulation while > 0 indicates up-regulation. Differential significance analysis was performed using the TukeyHSD function. *padj* value > 0.05 as shown no significance (ns.). **G** A boxplot diagram displaying expression levels of transcripts located in various genomic regions for switch genes with cncAPA. A log2 fold change (log2FC) < 0 indicates down-regulation while > 0 indicates up-regulation. Differential significance analysis was performed using the TukeyHSD function. *padj* value < 0.05 as shown *; *padj* value < 0.001 as shown ***; *padj* value > 0.05 as shown no significance (ns.)

To further elucidate the APA of switch genes (switch genes exhibit shifts in their poly(A) site usage) in response to HS, the QuantifyPoly(A) method was used (Ye et al. 2021). This method employs usage ratios of each PAC per gene to evaluate APA dynamics. First, we performed switch genes differential analysis between *siz1-2* mutant and Col-0 under RT or HS conditions. The results indicated that 126 and 292 switch genes with divergent poly(A) site usage exhibited differential expression in RT and HS, respectively (Fig. S4). Furthermore, overlap analysis revealed that only 72 of the 292 HS switch genes overlapped with those of the RT switch genes, suggesting that SIZ1 plays a regulatory role in APA switching under HS (Fig. S4). Next, we performed a comparative analysis of switch genes between HS and RT conditions in Col-0 and *siz1-2*, respectively. The results indicated that 334 and 289 switch genes with different poly(A) site usages were differentially expressed in Col-0 and *siz1-2*, respectively, in response to HS (Fig. 3A and Data S1 H, I). Furthermore, the deletion of *SIZ1* specially regulated 125 switch genes with different poly(A) site usages, suggesting that SIZ1 plays a role in determine the choice of poly(A) sites in HS response. We defined genes that preferentially utilize proximal poly(A) sites as “shortened switch genes”, while those that favor distal poly(A) sites were designated as “lengthened switch genes”. We quantified the APA dynamics among PACs located at the 3’UTR of an APA gene, referred to as canonical APA (canAPA). Thus, canAPA refers to genes with differences in the relative abundance of APA forms only located in the 3’UTR region. Following exposure to HS, 84 and 103 switch genes with canAPA and varying poly(A) site usages in their 3’UTR were found to be differentially expressed in Col-0 and *siz1-2*, respectively (Data S1J, K). Notably, there was an increase in the usage of distal 3’UTR sites by more switch genes (lengthened switch genes), especially in *siz1-2* (Fig. 3B, D). Compared to Col-0, the deletion of *SIZ1* did not significantly alter the expression levels of transcripts located at 3’UTR (Fig. 3F).

Additionally, we assessed the APA dynamics between two categories of PACs (canonical and non-canonical PACs in a gene), referred to as cncAPA. Non-canonical PACs were defined as those located within intragenic regions, excluding the 3’UTR. cncAPA refers to genes with differences in the relative abundance of canonical PACs in 3’UTR compared to non-canonical PACs. A total of 69 and 43 switch genes associated with cncAPA were identified in Col-0 and *siz1-2*, respectively (Data S1 L, M), indicating that more switch genes preferentially utilize sites within the 3’UTR (lengthened switch genes) (Fig. 3C, E). In addition, notably, expression levels of transcripts situated in 5’UTR and CDS decreased, while those in exon, ext_3’UTR, intergenic, and intron regions increased (Fig. 3G). However, compared to Col-0, the expression of transcripts located at 3’UTR, 5’UTR, and CDS significantly increased in *siz1-2* in response to HS. These findings demonstrate that APA operates across various genomic regions to modulate the expression of specific transcript forms mediated by SIZ1 during HS.

The usage of mRNA poly(A) sites is governed by the interaction between cis-elements on pre-mRNAs and a set of polyadenylation factors. To study these cis-elements, characterization of the single-nucleotide base compositions and poly(A) signals surrounding poly(A) sites was conducted in the Near Upstream Elements (NUEs) and Far Upstream Elements (FUEs). The poly(A) sites identified in canAPA and cncAPA were categorized into four groups: lengthened PACs in Col-0, shorten PACs in Col-0, lengthened PACs in *siz1-2*, and shortened PACs in *siz1-2*. In canAPA, compared to Col-0, the deletion of SIZ1 increased the uracil (U) bases and U-rich motifs in FUEs (Fig. S5, S6). In cncAPA, compared to the shortened PACs, more adenine (A) bases and A-rich signals in NUEs, and more U bases and U-rich motifs in FUEs were used in the lengthened PACs, especially in the Col-0 context (Fig. S5, S6). These analyses suggest that there are differences in signal usage between canonical and non-canonical poly(A) site usages.

### SIZ1 mediates the switching of poly(A) sites in genes associated with heat stress response

**Fig. 4:**
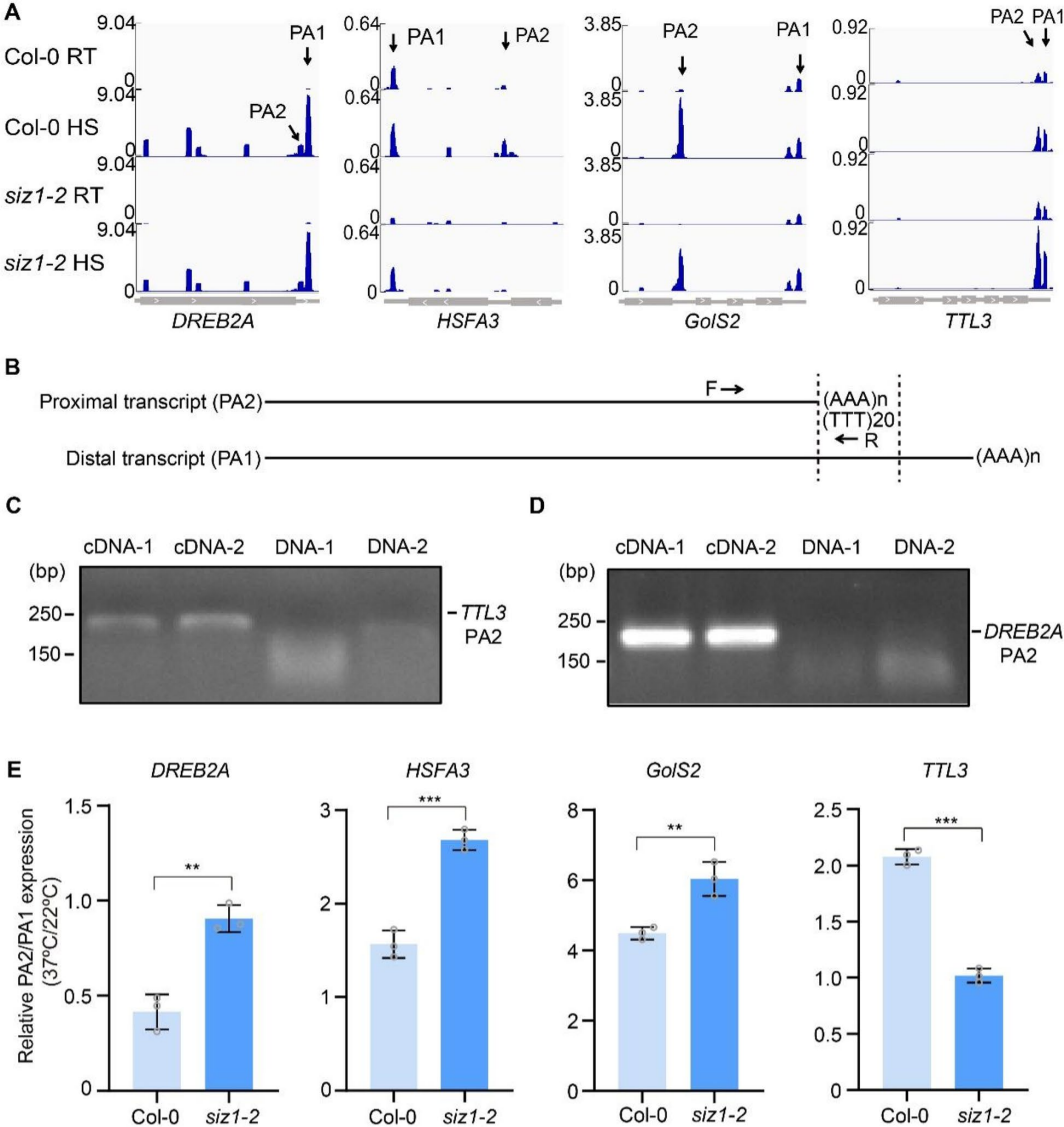
Verification of the expression ratios of APA transcripts for *DREB2A*, *HSFA3*, *GolS2*, and *TTL3*. **A** The sequencing coverage of related switch genes, as visualized using the Integrative Genomics Viewer. **B** A schematic representation illustrating the primer design for proximal transcripts. Reverse primers of proximal transcript incorporate a specific twelve-base sequence followed by twenty thymine bases (20 T), which complementarily pairs with poly(A). **C-D** Specific verification of PCR primers for *TTL3* and *DREB2A* proximal transcripts. Representative PCR images are displayed. Genomic DNA was used as a template in the control samples. cDNA and genomic DNA each have two templates: −1 and −2. The annealing temperature was set at 55 °C, with a total of 28 cycles was performed. **E** RT-qPCR analysis was conducted to determine the relative expression ratios of APA transcripts for *DREB2A*, *HSFA3*, *GolS2*, and *TTL3* in Col-0 and *siz1-2* backgrounds. The relative expression levels of different transcripts were calculated as the expression ratio of proximal transcript to distal transcript. The relative expression of indicated transcripts was calculated as the ratio of 37 °C/22 °C. after being normalized with the expression of the indicated transcript in Col-0 under 22 °C, the relative ratio shown in figure was calculated as the ratio of the relative expression of proximal transcript (37 °C/22 °C) to distal transcript (37 °C/22 °C). The ratio at RT (22 °C) was set as 1. *ACTIN2* served as an internal control. Data are mean ± SD from three technological replicates in a single experiment. Three biologically independent experiments yielded similar patterns. Significant differences were assessed using Student’s *t*-test (**p < 0.01, ***p < 0.001)

Based on the aforementioned data analysis, it served as an important resource for specific transcripts from APA in response to HS response for functional characterization. To further reveal the role of distinct transcript isoforms resulting from APA in response to HS, we visualized the PAT-seq coverage of the switch genes using Integrative Genomics Viewer. Among these, *DREB2A* and *HSFA3*, two genes encoding key transcription factors involved in HS response, were selected for analysis. The expression of transcripts with multiple poly(A) sites in *DREB2A* and *HSFA3* was induced to varying degrees by HS (Fig. 4A). In addition, we selected two HS-induced genes to characterize their novel functions: *GolS2*, a switch gene associated with cncAPA, and *TTL3*, a switch gene associated with canAPA. *GolS2* and *TTL3* were APA genes whose transcripts were also induced by HS (Fig. 4A). The expression data of PAC and genes for the above selected genes have been presented in Data S1N.

To verify the differential expression levels of these switch genes, we conducted RT-qPCR analysis. Previous studies have primarily focused on examining distal transcript expression levels within the 3’UTR due to specificity limitations associated with proximal reverse primers, because the transcript with the distal poly(A) site contains the sequence of the proximal poly(A) site. To accurately assess the expression levels of proximal transcripts, we developed a method for designing reverse primers that incorporate a specific twelve-base sequence followed by twenty thymine bases (20 T), which pairs complementarily with poly(A) (Fig. 4B). Initially, we evaluated the primer specificity for proximal transcripts located at the 3’UTR of *TTL3* and *DREB2A* using RT-PCR. The results demonstrated that the proximal transcripts of *TTL3* and *DREB2A* were amplified accurately and specifically via RT-PCR (Fig. 4C, D). Melt curve analysis showed a single peak, indicating that the primers exhibited high specificity (Fig. S7).

**Fig. 5:**
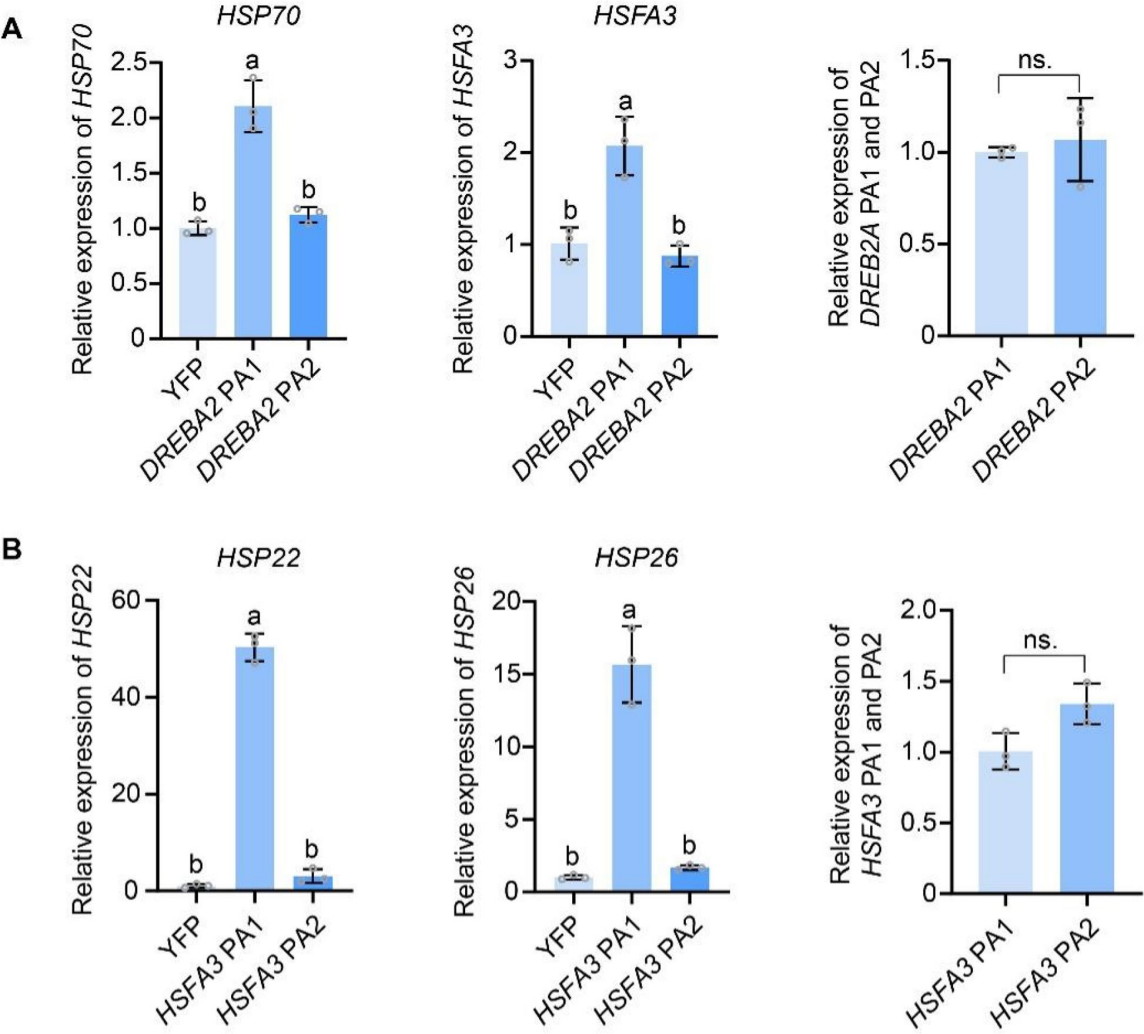
The function of different transcripts of *DREB2A* and *HSFA3* under HS. **A-B** Proximal (PA2) and distal isoform (PA1) were each constructed into a YFP expression vector, and expressed in wild-type cells. The cells were cultured overnight at 22 °C and subsequently subjected to HS treatment for 30 min. The YFP vector was used as a control. **A** The relative expression of *HSP70* and *HSFA3* in wild-type cells overexpressing PA1 and PA2 forms of *DREB2A* is shown in the left and middle graphs, respectively. The relative expression levels of different transcripts of overexpressed *DREB2A* are shown in the right graph. **B** The relative expression of *HSP22* and *HSP26* in wild-type cells overexpressing PA1 or PA2 forms of *HSFA3* is shown in the left and middle graphs, respectively. The relative expression levels of different transcripts of overexpressed *HSFA3* are shown in the right graph. Data are mean ± SD from three technological replicates in a single experiment. Three biologically independent experiments yielded similar patterns. Significant differences were analyzed using one-way ANOVA followed by Tukey’s multiple comparisons test (*p* < 0.05), and Student’s *t*-test (ns., *p* > 0.05)

Subsequently, RT-qPCR results indicated an increase in relative proximal/distal (PA2/PA1) expression ratios for *HSFA3*, *GolS2*, and *TTL3*, while a decrease was observed for *DREB2A* in response to HS (Fig. 4E), thereby confirming the impact of treatment time was extended to 4 days (the treatment time for HS tolerance phenotype), the relative ratio of PA2/PA1 in *HSFA3*, *GolS2*, *TTL3*, and *DREB2A* showed a similar trend of change compared with that after 30 min of HS (Fig. S8). Thus, the HS phenotype observed in our study may be directly caused by APA. Notably, compared to WT, the deficiency in *SIZ1* resulted in higher relative proximal/distal (PA2/PA1) expression ratios for *DREB2A*, *HSFA3*, and *GolS2* while a lower ratio for *TTL3* in response to HS*.* These findings suggest that SIZ1 mediates the poly(A) site usage of switch genes associated with HS. Because the *siz1-2* mutant is more sensitive to HS treatment. Consistently, the distal transcripts (PA1) of *DREB2A* promotes the expression of downstream genes, *HSP70* and *HSFA3*, and the distal transcripts (PA1) of *HSFA3* increased the expression levels of *HSP22* and *HSP26* (Fig. 5), suggesting that the distal transcript of *DREB2A* and *HSFA3* exert a specific role in conferring HS tolerance*.* Thus, the strategy that utilizes APA resources for identifying transcripts associated with plant tolerance to HS has proven effective, and possesses the potential for identifying novel transcripts.

### The distinct functions of different transcript isoforms of *GolS2* and *TTL3* in heat tolerance

**Fig. 6:**
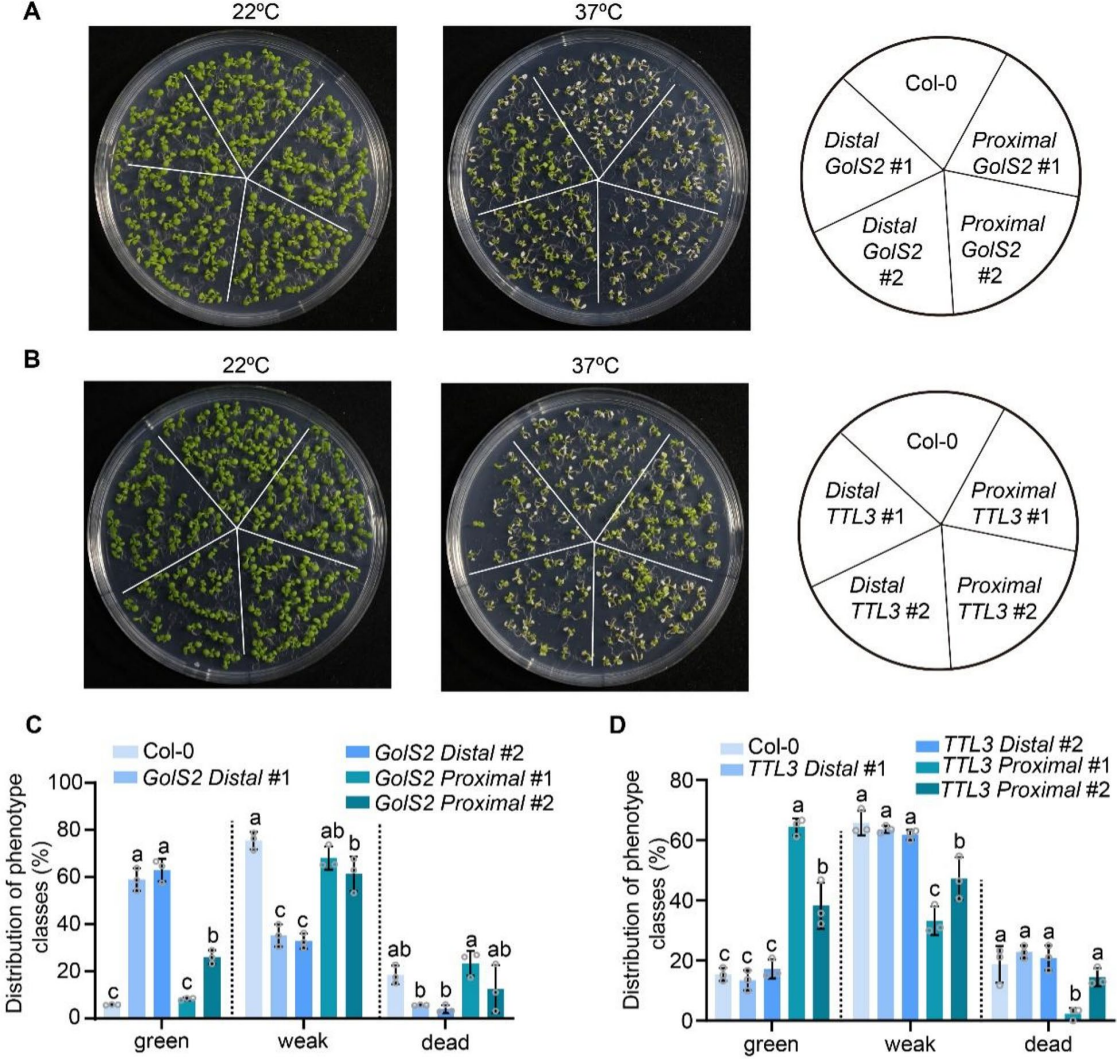
The effects of overexpressing either the proximal or distal variant of *GolS2* or *TTL3* on HS tolerance in Arabidopsis seedlings. **A-B** Representative phenotype images are displayed. Seven-day-old seedlings were subjected to a treatment at 37 °C for 4 days in an incubator maintained at 60% relative humidity, with seedlings remained at 22 °C serving as a control. The phenotype observed following recovery (3 d at 22 °C after treatment) is shown. **C-D** The percentages of green, weak (characterized by only two green leaves), and dead seedlings are shown. Data are mean ± SD from three biologically independent experiments. Three biologically independent experiments demonstrated consistent patterns. Significant differences were analyzed using one-way ANOVA followed by Tukey’s multiple comparisons test (*p* < 0.05)

Given that the genes whose expression is altered by HS may be associated with this stress condition, the specific transcripts of identified switch genes could differentially contribute to HS tolerance. Consequently, either the proximal or distal variant of *GolS2* and *TTL3* was overexpressed in a Col-0 background under the *UBQ10* promoter. Independent transgenic lines with comparable levels of transcripts were selected for phenotypic analysis under HS treatment (Fig. S9). Seven-day-old Arabidopsis wild-type seedlings and transgenic seedlings overexpressing the *GolS2* transcript isoforms were subjected to the treatment at 37 °C for 4 days, followed by a 3-day recovery period at 22 °C. Compared to the wild-type, overexpression of the distal transcript of *GolS2*, rather than its proximal transcript, significantly enhanced survival rates and reduced the percentages of weak seedlings (defined as those with only two green leaves) and dead seedlings under this HS condition (Fig. 6A, C). Conversely, the overexpression of the proximal transcript of *TTL3*, but not its distal transcript, markedly increased green survival rates in response to this HS treatment (Fig. 6B, D). In contrast, the overexpression of the distal transcript *TTL3* yielded results comparable to those of the wild type. These findings suggest that the distal transcript of *GolS2* and the proximal transcript of *TTL3* play a role in conferring HS tolerance. To further demonstrate the function of different transcripts of *GolS2* and *TTL3* under HS, either the proximal or distal isoform of *GolS2* and *TTL3* was overexpressed in *siz1-2* mutant cells to detect the expression of downstream genes, *HSP18* and *HSP22*. When the transcript levels of different transcripts of *GolS2* or *TTL3* were similar, we found that under HS, the PA1 isoform of *GolS2* could rescue the defective expression of *HSP18* and *HSP22* in the *siz1-2* mutant, whereas the PA2 isoform of *GolS2* could not (Fig. S10A). In contrast, the PA2 isoform of *TTL3* could rescue the defective expression of *HSP18* and *HSP22* in the *siz1-2* mutant under HS, while its PA1 isoform could not (Fig. S10B). These results are consistent with the function of *TTL3* and *GolS2* different transcripts in wild type in response to HS.

**Fig. 7:**
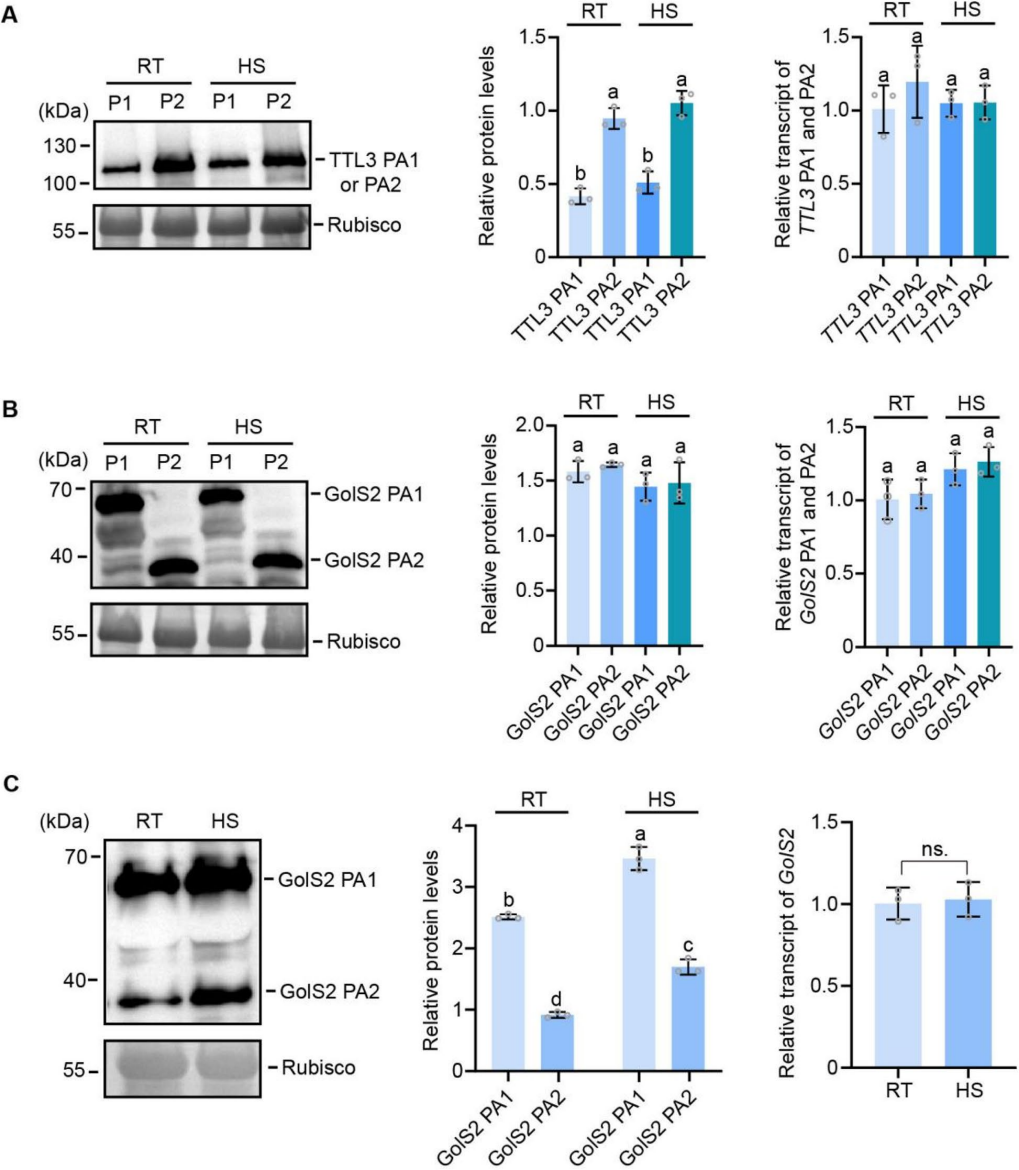
The effect of different transcripts of *TTL3* and *GolS2* on their protein expression. **A-B** Different transcript isoforms were fused with GFP and overexpressed in plant cells for protein and transcript analyses. Protein levels were analyzed by immunoblotting using anti-GFP antibody, as shown in the left graph; quantitative analysis of the relative protein levels of TTL3 and GolS2 is presented in the middle graph. The relative transcript levels of overexpressed PA1 and PA2 in wild-type cells are shown in the right graph. **C** Characterization of *GolS2* protein isoforms produced from its genome region. The genome region of *GolS2* was fused to GFP for overexpression in plant cells. Protein levels were analyzed by immunoblotting using anti-GFP antibody, as shown in the left graph; quantitative analysis of the relative protein levels of different GolS2 isoform is shown in the middle graph. The relative expression levels of overexpressed *GolS2* in wild-type cells are shown in the right graph. RT, cells overnight at 22 °C. HS, cells overnight at 22 °C and then subjected to HS treatment for 30 min. Data are mean ± SD from three technological replicates in a single experiment. Three biologically independent experiments yielded similar patterns. Significant differences were analyzed using one-way ANOVA followed by Tukey’s multiple comparisons test (*p* < 0.05), and Student’s *t*-test (ns., *p* > 0.05)

To study the impact of different transcripts of *TTL3* and *GolS2* on protein expression, we analyzed the levels of proteins encoded by different transcripts of *TTL3* and *GolS2* when their transcript levels were similar. The data indicated that the level of TTL3 protein produced by PA2 isoform was higher that of PA1 isoform (Fig. 7A), consistent with the result that overexpression of the PA2 isoform of *TTL3* enhances HS tolerance. For GolS2, the proteins encoded by its two distinct transcripts exhibit comparable abundance when their transcript levels are similar (Fig. 7B). Further immunoblotting data confirmed that the *GolS2* gene encodes two protein isoforms with distinct sizes (Fig. 7C). Although sequencing data showed that HS significantly upregulates the expression of the PA2 transcript of *GolS2*, the protein abundance of PA1 isoform was higher than that of PA2 isoform. This conclusion is consistent with the phenotype wherein overexpression of the *GolS2* PA1 isoform enhances HS tolerance. Based on the above evidence, it is supported that the current strategy is efficient to identify specific transcripts arising from APA for regulating HS responses.

## Discussion

Following global warming, HS has emerged as a critical environmental challenge with significant impacts on plant health. Previous studies have focused on the transcriptional networks involved in plant HS responses, but RNA processing mechanisms such as APA can produce distinct transcript isoforms that may confer varied functions. However, the function of APA in this process remains unclear. Furthermore, the SUMO E3 ligase SIZ1 has been identified as a regulator of transcriptional responses to HS in Arabidopsis, but existing reports have predominantly concentrated on its role in regulating SUMOylation substrates related to heat-responsive transcription (Han et al. 2021; Huang et al. 2023). Given that SIZ1 regulates numerous substrates and may influence gene expression at multiple levels, elucidating the potential connection between SIZ1 and APA in HS response is important for uncovering the regulatory mechanisms underlying plant tolerance to HS. Notably, our recent research indicated that SIZ1 regulates APA during thermomorphogenesis under warm ambient temperatures (Yu et al. 2024). Nevertheless, since thermomorphogenesis and HS response are distinct biological processes, it remains unknown whether SIZ1 plays a role in regulating APA in plant HS responses. In the current study, our mRNA 3’ end PAT-seq transcriptome analysis demonstrates that SIZ1 mediated APA for the generation of specific transcript isoforms with diverse functional implications in Arabidopsis.

Previous research showed that the accumulation of non-canonical mRNA is promoted under stress conditions (Shaul 2015). In this study, we observed a decrease in the distribution of DE-PACs in 3’UTR and exon regions, while an increase in intron and intergenic regions. Furthermore, during cncAPA switch events, the expression levels of non-canonical isoforms, including exon, intron, and intergenic regions were increased. Our results indicated that HS significantly increased the accumulation of non-canonical site usage. This finding aligns with previous research demonstrating that stress conditions lead to an increase in non-canonical isoforms (Chakrabarti et al. 2020; de Lorenzo et al. 2017). In addition, during canAPA switch events, the deficiency of *SIZ1* resulted in more genes exhibiting lengthening, suggesting that more genes prefer to use distal 3’UTR sites under HS. Previous research reported that high salt stress led to more gene 3’UTR lengthening through increasing the usage of distal poly(A) sites (Wang et al. 2023). Consistently, Liu et al. used GRO-seq and RNA-seq to reveal transcriptional readthrough events under HS (Liu et al. 2021). Thus, it may be a universal phenomenon that stress causes plant mRNA to utilize distal sites in 3’UTR.

In plants, APA events are tightly associated with environmental responses, including oxidative stress, hypoxia, heat exposure, dehydration, drought, salinity, and heavy metal toxicity (Cao et al. 2019; Chakrabarti et al. 2020; de Lorenzo et al. 2017; Ma et al. 2022; Sun et al. 2017; Wang et al. 2023; Zhang et al. 2008). Analysis of APA from rice RNA-seq data revealed that heat shock protein coding genes exhibit dynamic APA in response to HS (Ye et al. 2019). However, the functions of specific transcripts with distinct poly(A) sites in HS response have yet to be elucidated in plants. Based on the different profiles of APA in the wild-type and *siz1-2* plants, we identified two known HS-induced genes: *DREB2A* and *HSFA3*. The distal 3’UTR PA1 of *DREB2A* and *HSFA3* promotes the expression of downstream genes. Importantly, we identified two unknown HS-induced genes: *GolS2*, a switch gene associated with cncAPA, and *TTL3*, a switch gene associated with canAPA. GolS2 is a key enzyme in the biosynthetic pathway of raffinose family oligosaccharides. Previous studies have demonstrated that sugar-modulated gene expression is closely associated with the maintenance of ROS balance, indicating that *GolS* genes are implicated in numerous physiological responses (ElSayed et al. 2014; Taji et al. 2002). TTL proteins exhibit a conserved modular architecture consisting of six tetratricopeptide repeats. TTL proteins facilitate the assembly of multiprotein complexes and are involved in responses to osmotic stress (Lakhssassi et al. 2012; Rosado et al. 2006). However, the function of the above two genes in HS responses have not been reported. In this study, the phenotypic data indicated that the overexpression of the distal transcript of *GolS2* or the proximal transcript of *TTL3* enhances heat tolerance. These phenotypes align with the observation in *siz1-2*, where an elevated relative proximal/distal (PA2/PA1) expression ratio for *GolS2* are noted, while a reduced ratio is observed for *TTL3*. In addition, we found that that overexpression of *TTL3* proximal (PA2) isoform increased the expression of downstream genes, *HSP18* and *HSP22* under HS, which was similar to the function of the *TTL3* CDS (Fig. S11), supporting that the function of APA. This supports the approach of identifying specific transcripts associated with HS tolerance by utilizing the APA data from *siz1-2* mutant plants.

The switching of poly(A) sites may regulate RNA stability or protein production (Tian and Manley 2017). Here, we found that the PA2 isoform of *TTL3* transcript produces more protein compared with PA1, which aligns with the phenotype where overexpression of PA2 enhances HS tolerance. Compared to wild type, sequencing data showed that *TTL3* PA1 and PA2 transcripts were simultaneously up-regulated in *siz1-2* mutant under HS, but the *siz1-2* mutant was more sensitive to HS. We speculate that *siz1-2* phenotype under heat stress may be related to the ratio of PA1/PA2. In addition, the level of GolS2 protein produced by PA1 was higher than that of PA2, and HS promoted the level of GolS2 PA1-coding protein, which aligns with the phenotype where overexpression of PA1 enhances HS tolerance. However, sequencing data showed the transcript levels of *GolS2* PA2 significantly increased under HS in *siz1-2*. Thus, the relationship of between the transcription and protein levels of different transcripts in response to HS need to be further elucidated.

Notably, the HS-induced APA is precisely regulated by SIZ1, likely due to its SUMO ligase activity. Our recent research demonstrated that SIZ1 enhances the SUMOylation of CPSF100, a critical subunit of the cleavage and polyadenylation specificity factor (CPSF) complex involved in APA during thermomorphogenesis (Yu et al. 2024). Here, we found that SIZ1 also mediates the SUMOylation of CPSF100 under HS, and HS enhances its SUMOylation (Fig. S12), suggesting that SIZ1 may mediate the SUMOylation of polyadenylation factors to facilitate APA, thereby contributing to the generation of specific transcript isoforms with diverse functional implications during HS response in Arabidopsis.

## Conclusion

Our findings demonstrate that SIZ1-mediated HS response alters the utilization of switch poly(A) sites. These changes facilitate the generation of specific transcript isoforms with diverse functions in Arabidopsis. Based on phenotypic data derived from the overexpression of distinct transcripts of *GolS2* and *TTL3*, it is suggested that the roles played by distal and proximal transcripts may vary among different genes, but detailed mechanisms underlying these differences remain to be elucidated. Importantly, this genome-wide APA resource based on SIZ1 can be utilized for identifying novel transcripts associated with plant tolerance to HS. Given that the APA mechanism is conserved across eukaryotic cells, this approach holds potential applicability in other plant species and could significantly contribute to enhancing stress tolerance in crops.

## Materials and methods

### Plant materials and growth conditions

The plant materials used in this study were *Arabidopsis thaliana *(L.)* Heynhold* ecotype Col-0. The seeds of *siz1-2* (SALK_065397) were obtained from the Arabidopsis Stock Center. The seeds were sterilized for 5 min in a solution of 2.5% NaClO containing 0.05% Triton X-100, followed by five rinses with sterile water. Subsequently, the seeds were sown on Murashige and Skoog medium supplemented with 1.5% (w/v) sucrose, 0.8% (w/v) agar, 0.05% MES, and pH 5.7, and stratified at 4 °C for 3 days. Plants were cultivated under long-day conditions (16 h light/8 h dark) at 22 °C. For PAT-seq library construction and RT-qPCR analysis, samples were collected after 3 h light treatment. For phenotypic analysis under HS treatment, 7-day-old seedlings were incubated at 37 °C for 4 days in an incubator before being transferred back to growth conditions at 22 °C growth for an additional 3 days. The phenotypic characteristics was observed and photographed, and the survival ratio of the seedlings was subsequently calculated.

### Generation of transgenic plants

To generate plants overexpressing either proximal or distal *GolS2* and *TTL3* in the Col-0 background, the respective proximal or distal version of *GolS2* and *TTL3* was cloned into the *pCambia* vector under the control of a *UBQ10* promoter. Transgenic plants were generated by Agrobacterium-mediated transformation using the floral-dip method (Clough and Bent 1998). Two independent homozygous transgenic lines for overexpression of each transcript were used for phenotypic analysis.

### PAT-seq library construction and sequencing

For the construction of PAT-seq libraries, 7-day-old seedlings were subjected to HS treatment at 37 °C for 30 min in a circulating water bath, while control samples were maintained at 22 °C for growth as RT conditions. Total RNA was extracted from three biologically independent replicates using the TRIzol reagent (Invitrogen), and any potential DNA contamination was eliminated with DNase I (Takara), followed by column-based RNA purification. Subsequently, 2 µg of total RNA was used to prepare PAT-seq libraries according to previously established protocols (Yu et al. 2019). The quality and quantity of the libraries were assessed using Qubit 2.0, Agilent Bioanalyzer 2100, and qPCR. The KAPA Library Quantification Kit is used for the qPCR accurate quantification of libraries. Illumina sequencing libraries are flanked by specific adapter sequences (P5 and P7 oligonucleotides) that bind to the flow cell during sequencing. The KAPA kit uses universal primers complementary to these conserved P5 and P7 adapter regions. These primers specifically amplify only the library molecules. Finally, sequencing was performed on the Illumina HiSeq 2500 platform at the facility located in the College of the Environment and Ecology, Xiamen University. The single-end poly(A) reads were generated for subsequent bioinformatics analysis.

### Poly(A) site extraction

Poly(A) sites were obtained following the previously described pipelines (Wu et al. 2011). The FASTX Toolkit was used to remove low-quality reads (version 0.0.14, parameters, fastq_quality_filter with -q 10 -p 50 -v -Q 33). Clean reads were mapped to the Arabidopsis reference genome (The Arabidopsis Information Resource 10th annotation; www.Arabidopsis.org) using Bowtie2 software (version 2.1.0, parameters “-L 25, -N 0, -i S,1,1.15–no-unal”). Internal priming events were filtered out. Poly(A) sites were extracted using an R package (Ye et al. 2021).

### Differential expression analysis

The DESeq2 package (version 1.42.0) was used to normalize reads and compare differential expression of genes and poly(A) sites between RT and HS. An adjusted *p* value < 0.05 was used to identify significant DE-PACs. DE-PACs belonging to APA genes were classified as DE-APA genes. All PATs of the genes were summed to represent gene expression levels. Similarly, DE genes were determined using the DESeq2 package. An adjusted *p* value < 0.05 was used to identify significant DE genes.

### APA dynamics identification

A weighted density peak clustering model was employed to analyze the dynamics of APA, following the methods previously described (Ye et al. 2021). *P* values were averaged to assess the significance of differences, with a cutoff of* p* < 0.05 indicating statistical significance.

### Gene Ontology (GO) and Kyoto Encyclopedia of Genes and Genomes (KEGG) enrichment analyses

GO analysis was conducted using Agrigo2 (v.2.0). DE-APA genes were submitted to Agrigo2 (http://systemsbiology.cau.edu.cn/agriGOv2/) for GO enrichment analysis. Singular enrichment analyses were performed, utilizing the Arabidopsis Information Resource 10th annotation as the background. A false discovery rate corrected *p* value of < 0.05 was applied as the cutoff for significant GO categories. KEGG enrichment analysis was performed using the KOBAS platform (Bu et al. 2021). Arabidopsis candidate gene IDs were converted to Entrez Gene IDs using DAVID. DE-APA genes were submitted to KOBAS (http://bioinfo.org/kobas/annotate/). Only pathways with a significance level of *p* value < 0.05 are shown.

### Poly(A) signal analysis

Sequences were extracted from switch genes located near poly(A) sites, spanning from 300 nucleotides (nts) upstream to 100 nts downstream. The canonical AAUAAA signal, along with its 1-nt variants, was analyzed in the NUE regions between −10 and −35 nts upstream of the poly(A) sites (Lin et al. 2017). Multiple Em for Motif Elicitation (MEME) was employed to enrich the poly(A) signals in the FUE regions, which are located from −150 to −35 nts relative to the poly(A) site (Bailey et al. 2009; Martin et al. 2012).

### RT-qPCR analysis

Aapproximately 1 microgram of total RNA, free from DNA contamination, was reverse transcribed using oligo(dT)18 primers with reverse transcriptase. Further qPCR was conducted on a Bio-Rad CFX96 Real Time system (Bio-Rad) with SYBR Premix ExTaqII fluorescent dye. *ACTIN2* served as the reference gene. All primer sequences used in this study are listed in Data S1O.

For the analysis of the expression ratios of APA transcripts for *DREB2A*, *HSFA3*, *GolS2*, and *TTL3*, 7-day-old wild-type seedlings were incubated at 37 °C for 4 days in an incubator or for 30 min in a circulating water bath. Then total RNA was extracted, followed by RT-qPCR. The relative expression of indicated transcripts was calculated as the ratio of 37 °C/22 °C. After being normalized with the expression of the indicated transcript in Col-0 under 22 °C conditions, the relative ratio shown in figure was calculated as the ratio of the relative expression of proximal transcript (37 °C/22 °C) to distal transcript (37 °C/22 °C).

For analyzing the effects of distinct *DREB2A* and *HSFA3* transcript isoforms on downstream gene expression under heat stress, Different transcript isoforms were overexpressed in wild-type protoplasts. After overnight incubation, total RNA was extracted for RT-qPCR analysis.

### SUMOylation assays

For the in vivo SUMOylation assay, experimental procedure was accomplished as previously described (Yu et al. 2024). CPSF100-GFP was co-expressed with Myc-SUMO1GG in wild type or *siz1-2* mutant protoplasts. After overnight incubation, samples were subjected to 37ºC for 30 min in a circulating water bath as HS treatment. Cells were harvested, and total proteins were extracted in the immunoprecipitation (IP) buffer. The protein lysates were then immunoblotted with anti-GFP and anti-Myc antibodies.

## Supplementary Information

Supplementary Material 1.

Supplementary Material 2.

## Data Availability

PAT-seq data for this study have been deposited to the NCBI Sequence Read Archive (SRA; http://www.ncbi.nlm.nih.gov/sra/) under the Bioproject accession numbers: PRJNA1195659.

## Abbreviations

HS: Heat stress
RT: Room temperature
GolS2: Galactinol synthase enzyme
TTL3: Tetratricopeptide repeat (TPR) like 3
PAT-seq: Poly(A) tag sequencing
Poly(A): Polyadenylation
APA: Alternative polyadenylation
PAC: Poly(A) site clusters
NUE: Near Up Element
FUE: Far Upstream Element
CDS: Coding sequence
UTR: Untranslated region
CanAPA: Canonical APA
CncAPA: Canonical and non-canonical PACs
DE-PAC: Differentially expressed PAC
GO: Gene ontology
