# Supplementary material for: Identification of specific transcripts for plant heat stress tolerance through genome-wide analysis of SIZ1-dependent alternative polyadenylation
Source: Stress Biol. 2026 Aug 18;6(1):61. doi: 10.1007/s44154-026-00340-2 (PMC13481651; doi:10.1007/s44154-026-00340-2)
Supplement: Supplementary file 1 [file 44154_2026_340_MOESM2_ESM.pdf]

Identification of specific transcripts for plant heat stress tolerance through genome-wide analysis of SIZ1-dependent alternative polyadenylation

Jun Wang[1], Xiujuan Wu[1], Zhou Zhou[1], Sitong Zheng[1], Minghui Hu[1], Yincui Xiao[1], Leqian Shi[1], Cheng Zhang[1], Jiamei Li[1], Chengwei Yang[1], Jianbin Lai[1,*], Danlu Han[1,*] and Zhibo Yu[1,*]

[1] Guangdong Provincial Key Laboratory of Biotechnology for Plant Development, School of Life Science, South China Normal University, Guangzhou 510631, China

*Correspondence: Zhibo Yu (yuzhibo@scnu.edu.cn), Danlu Han (danluhan@m.scnu.edu.cn), Jianbin Lai (20141062@m.scnu.edu.cn)

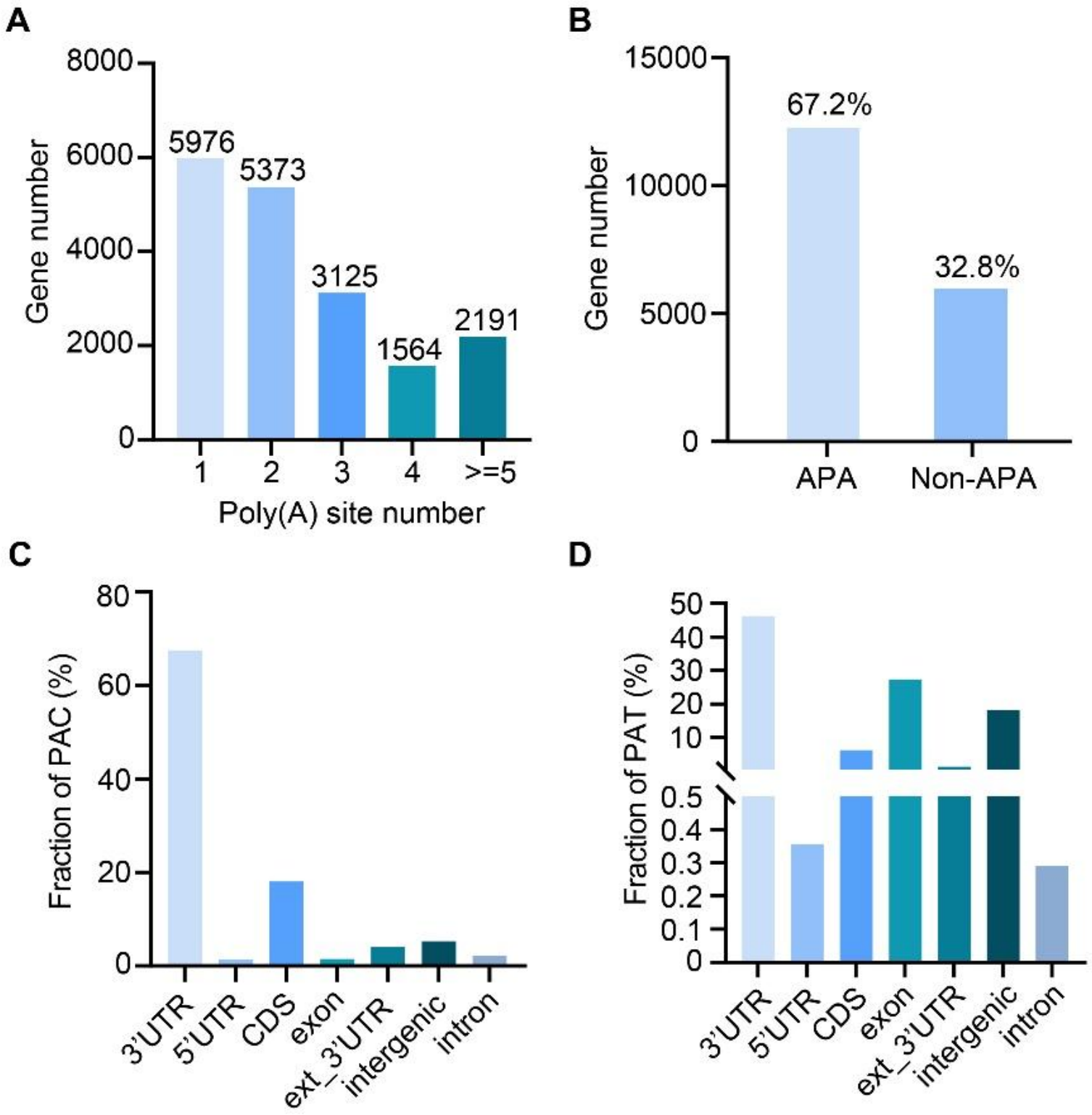

Fig. S1 Analysis of the number of APA genes and distribution of Poly(A) clusters (PACs) and Poly(A) tags (PATs). **A** The numbers of genes with varying numbers of poly(A) sites. **B** The number and ratio of APA and non-APA genes. APA genes, defined as those with two or more poly(A) sites, account for 67.2%. Non-APA genes, which contain only one poly(A) site, account for 32.8%. **C-D** The distribution of PAC and PAT across different genomic regions. PAC is defined as lying within 24 nucleotides of adjacent sites. UTR, untranslated region. CDS, coding sequence. ext_3'UTR refers to genes with annotated 3'UTRs that have been extended by 120 nucleotides, whereas genes lacking annotated 3'UTRs are extended by 338 nucleotides.

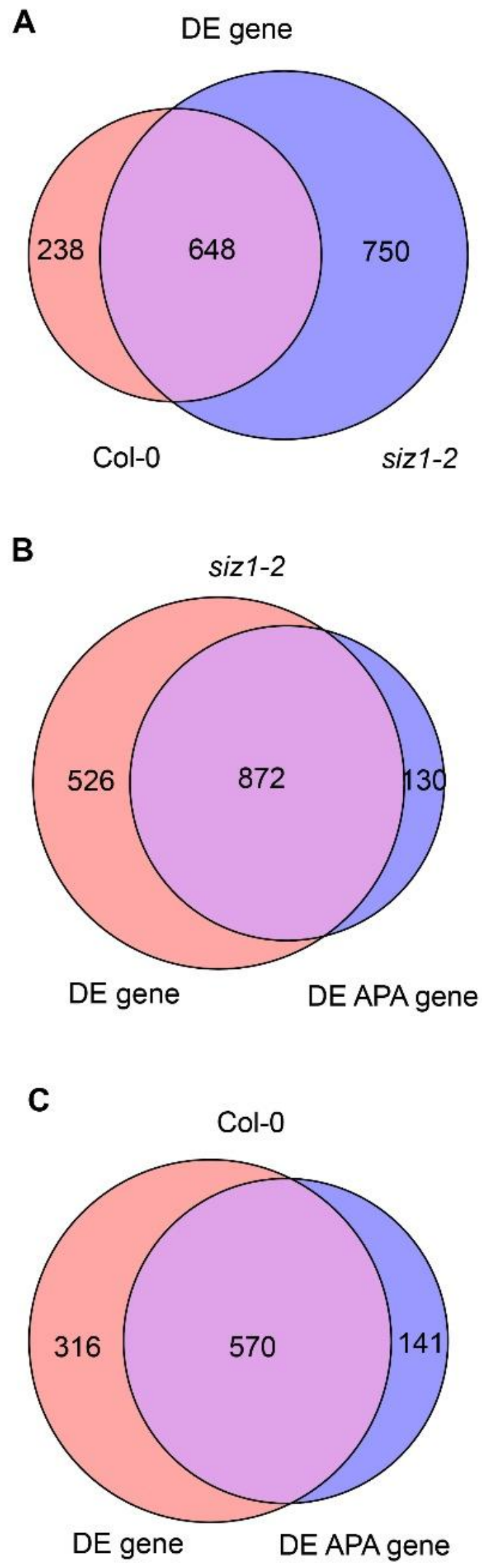

Fig. S2 Analysis of differentially expressed genes (DE genes) in response to heat treatments. An adjusted *p*-value < 0.05 was used to identify significant DE genes. **A** Venn diagram analysis of DE genes between Col-0 and *siz1-2* genotypes. **B-C** Overlaps of DE-APA genes and DE genes in Col-0 and *siz1-2*, respectively. DE-PAC containing genes were termed as DE-PAC genes, and DE-PAC genes with multiple poly(A) sites were classified as DE-APA genes.

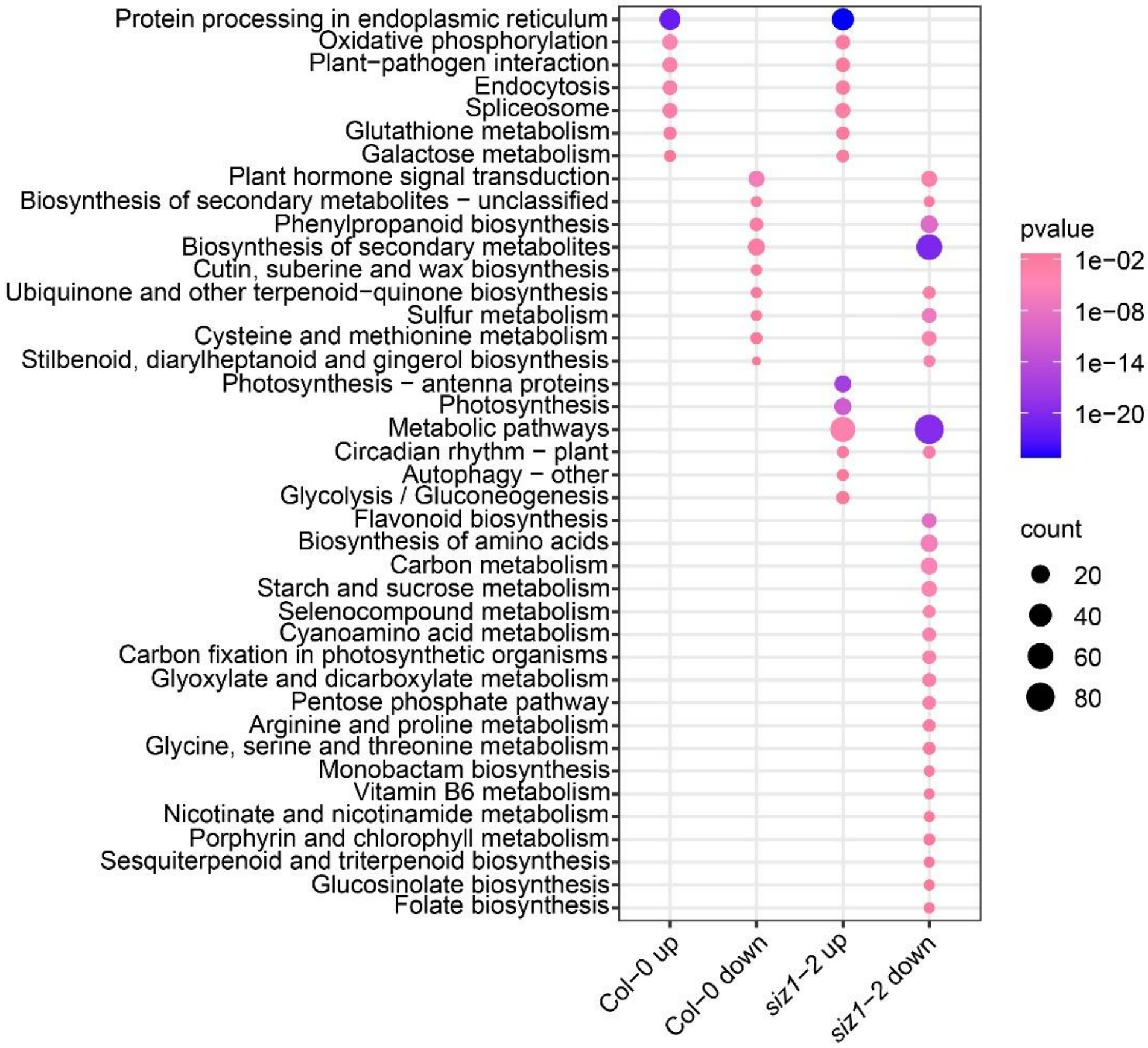

Fig. S3 The KEGG pathway enrichment analysis for down-regulated DE-APA genes and up-regulated DE-APA genes in Col-0 and *siz1-2* backgrounds. Only pathways with a significance level of $p < 0.05$ are shown.

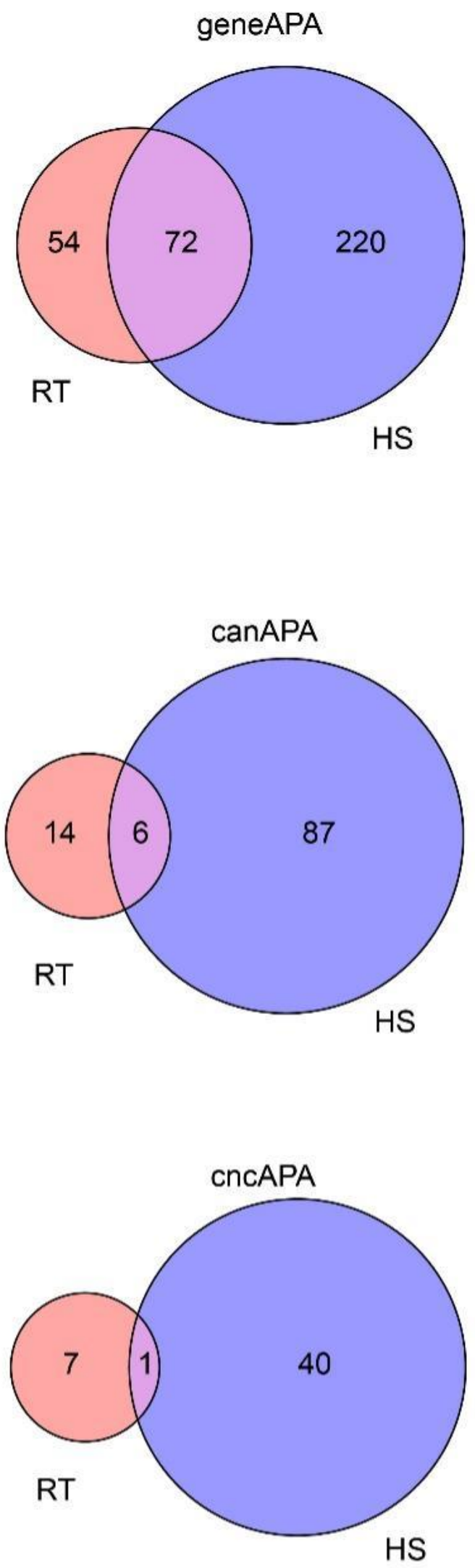

Fig. S4 Switch genes differential analysis between *siz1-2* mutant and Col-0 under room temperature (RT) or heat stress (HS) conditions. A *p*-value of less than 0.05 was considered statistically significant for poly(A) sites usage of switch genes. GeneAPA quantifies APA dynamics among all PACs associated with a specific gene, treating with each PAC equally. CanAPA quantifies APA dynamics among canonical PACs located at the 3'UTR of a specific gene. CncAPA quantifies APA dynamics between two categories of PACs, including both canonical and non-canonical PACs in a gene. Non-canonical PACs refer to those located in intragenic regions excluding the 3'UTR.

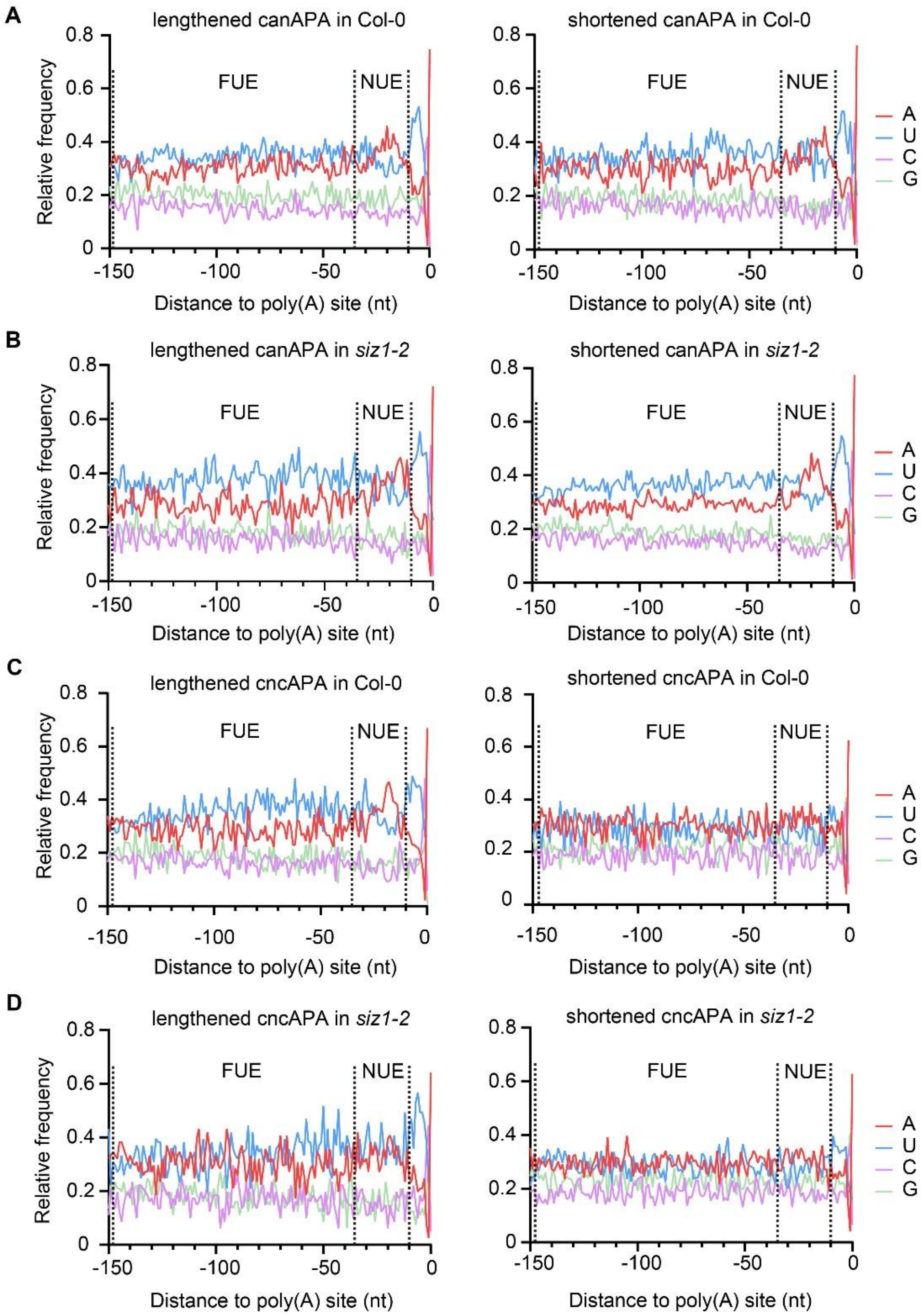

Fig. S5 Position-by-position analysis of average base composition in upstream of poly(A) sites. **A-B** Shortened and lengthened switch genes with canAPA in Col-0 and *siz1-2*. **C-D** Shortened and lengthened witch genes with cncAPA in Col-0 and *siz1-2*. The y-axis represents the fraction of nucleotide composition at the corresponding x-axis locations.

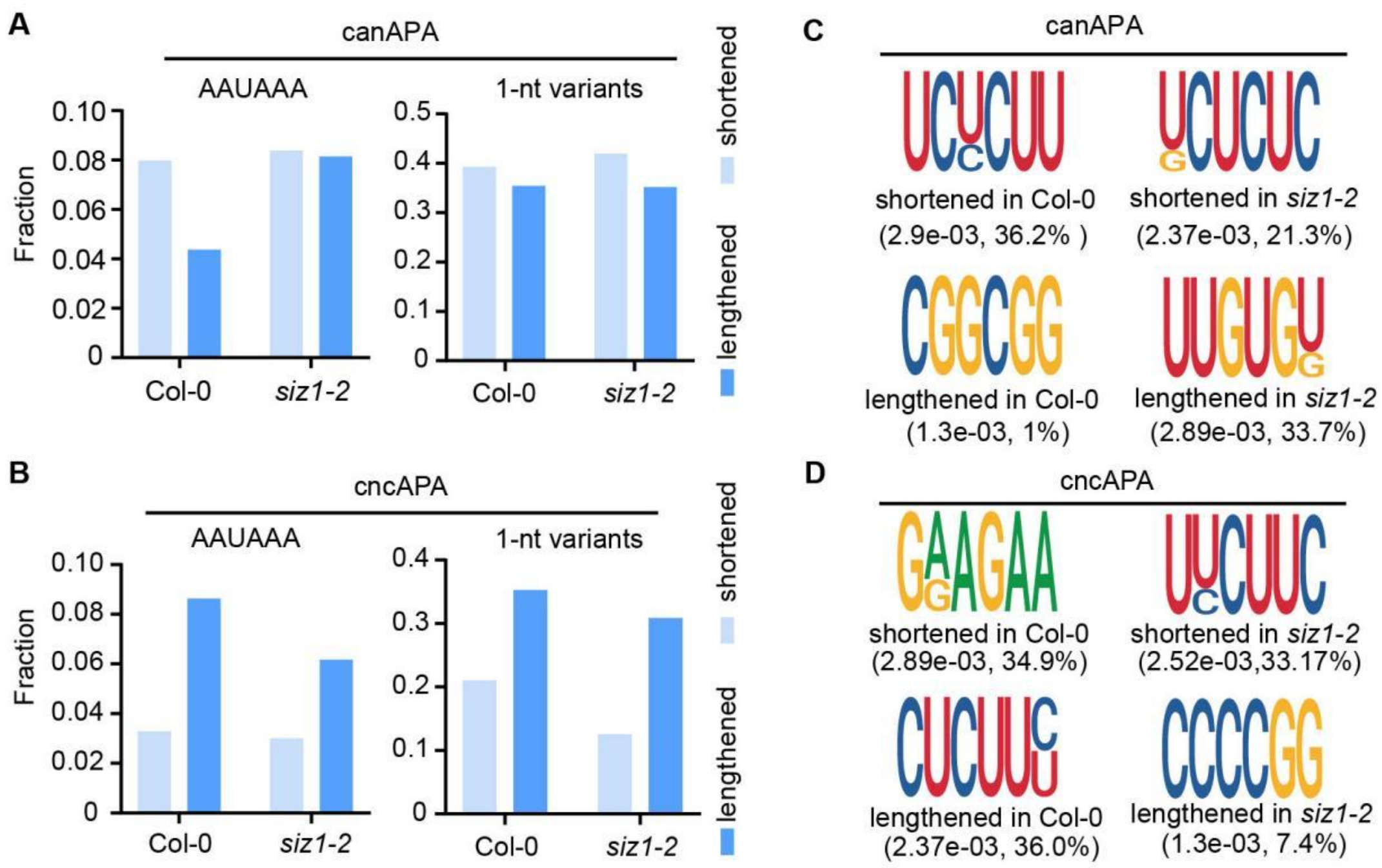

Fig. S6 The usage of poly(A) signals in the lengthened and shortened transcripts in response to HS. **A-B** The AAUAAA signal and its 1-nt variants in NUEs for canAPA and cncAPA. **C-D** Poly(A) signal motifs in FUEs identified by MEME. *P* value and percentage (transcripts of enriched motifs/total transcripts) are shown in the bottom of each motif.

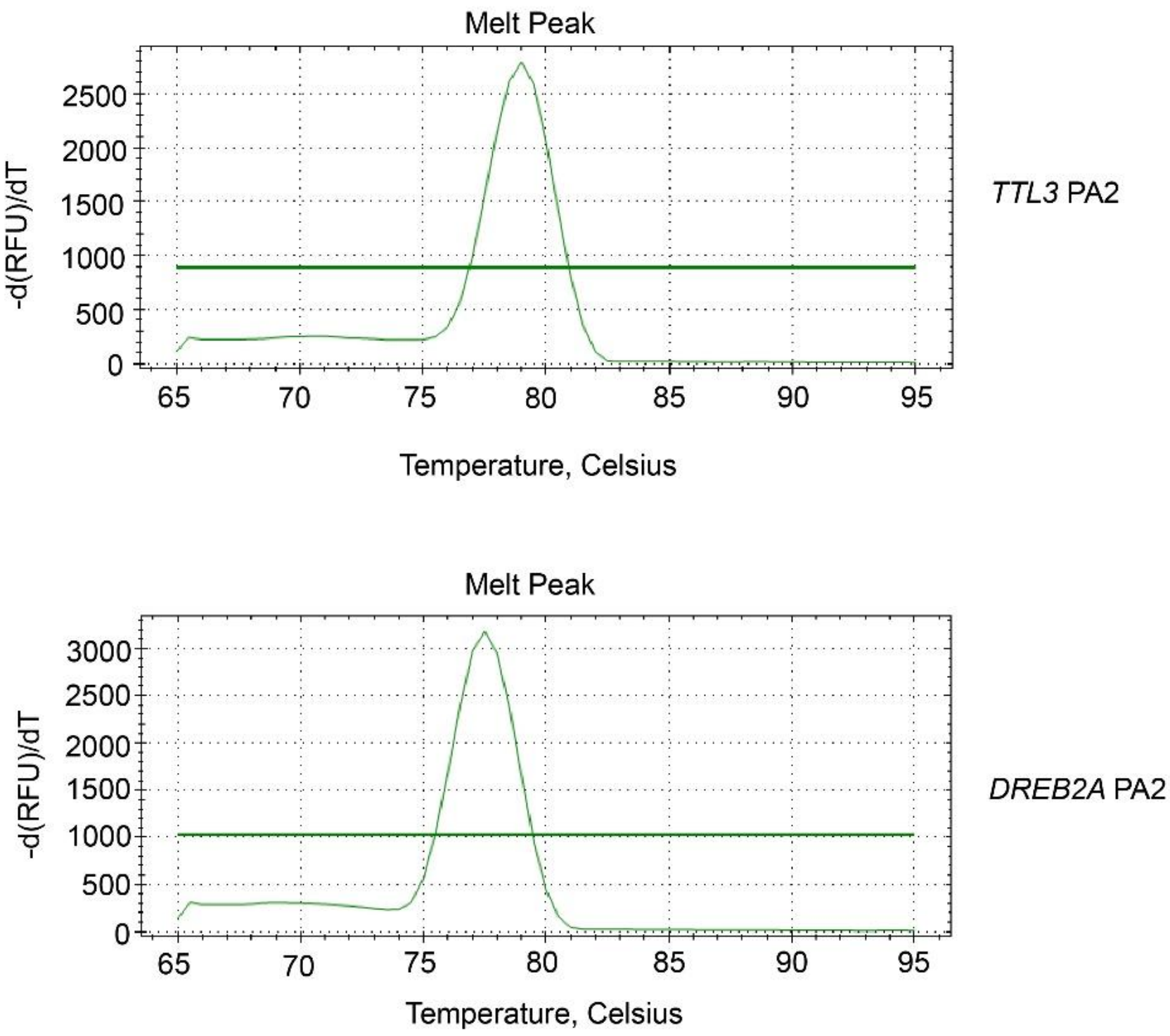

Fig. S7 Melt curve analysis for the proximal transcript primers targeting *TTL3* and *DREB2A* for RT-qPCR. The annealing temperature used for this analysis was set at 55°C.

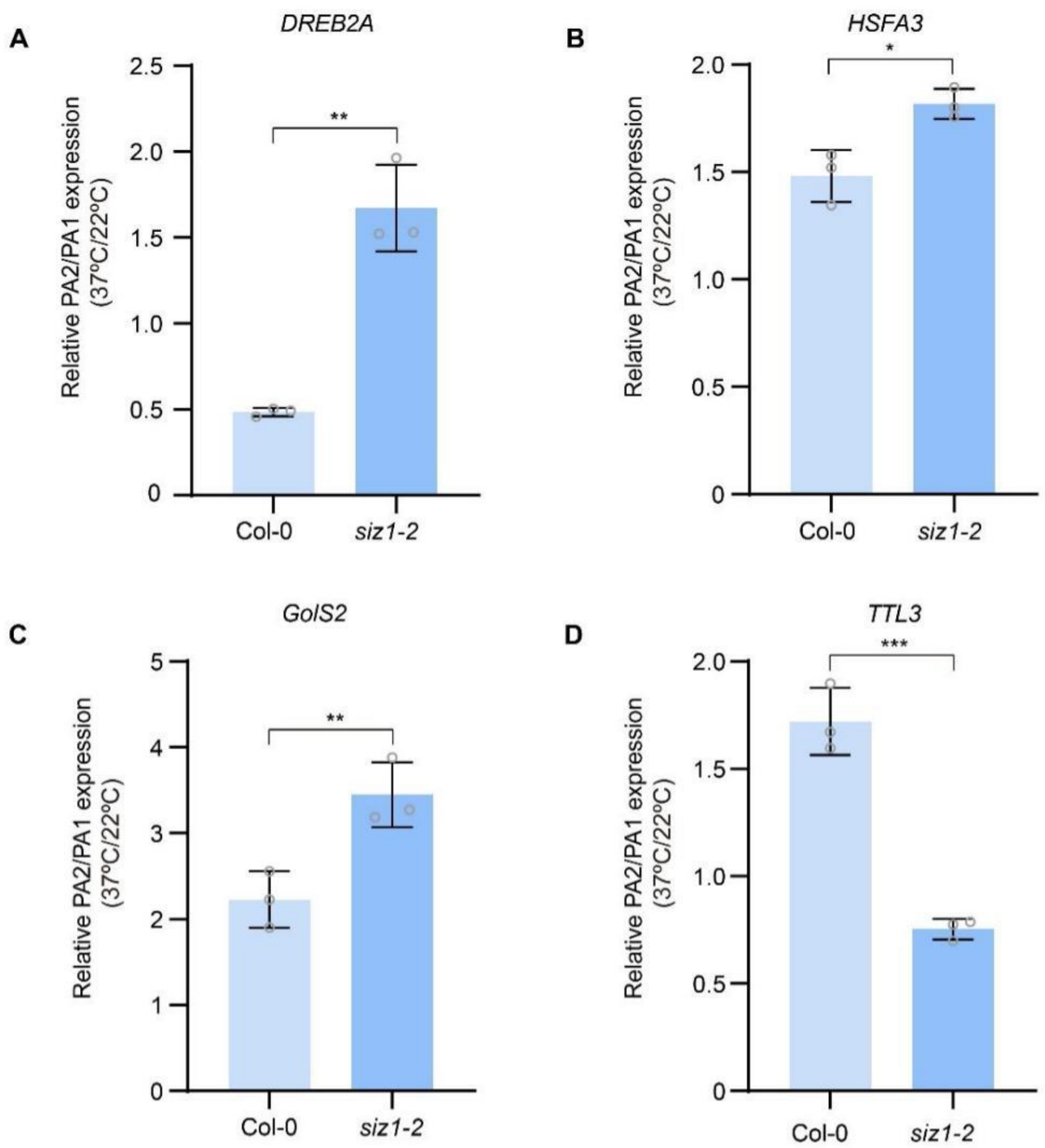

Fig. S8 The analysis of the expression ratios of APA transcripts for *DREB2A*, *HSFA3*, *GolS2*, and *TTL3* with or without heat treatment in Col-0 and *siz-2* plants. RT-qPCR analysis was performed on samples subjected to heat treatment for 4 days. The relative expression ratios of APA transcripts for *DREB2A* **A**, *HSFA3* **B**, *GolS2* **C**, and *TTL3* **D** in Col-0 and *siz1-2* backgrounds were determined. The relative expression levels of different transcripts were calculated as the expression ratio of proximal transcript to distal transcript. The relative expression of indicated transcripts was calculated as the ratio of 37°C/22°C. After being normalized with the expression of the indicated transcript in Col-0 under 22°C conditions, the relative ratio shown in figure was calculated as the ratio of the relative expression of proximal transcript (37°C/22°C) to distal transcript (37°C/22°C). *ACTIN2* served as an internal control. Data are mean ± SD from three technological replicates in a single experiment. Three biologically independent experiments yielded similar patterns. Significant differences were assessed using Student's *t*-test (*$p < 0.05$, **$p < 0.01$, ***$p < 0.001$).

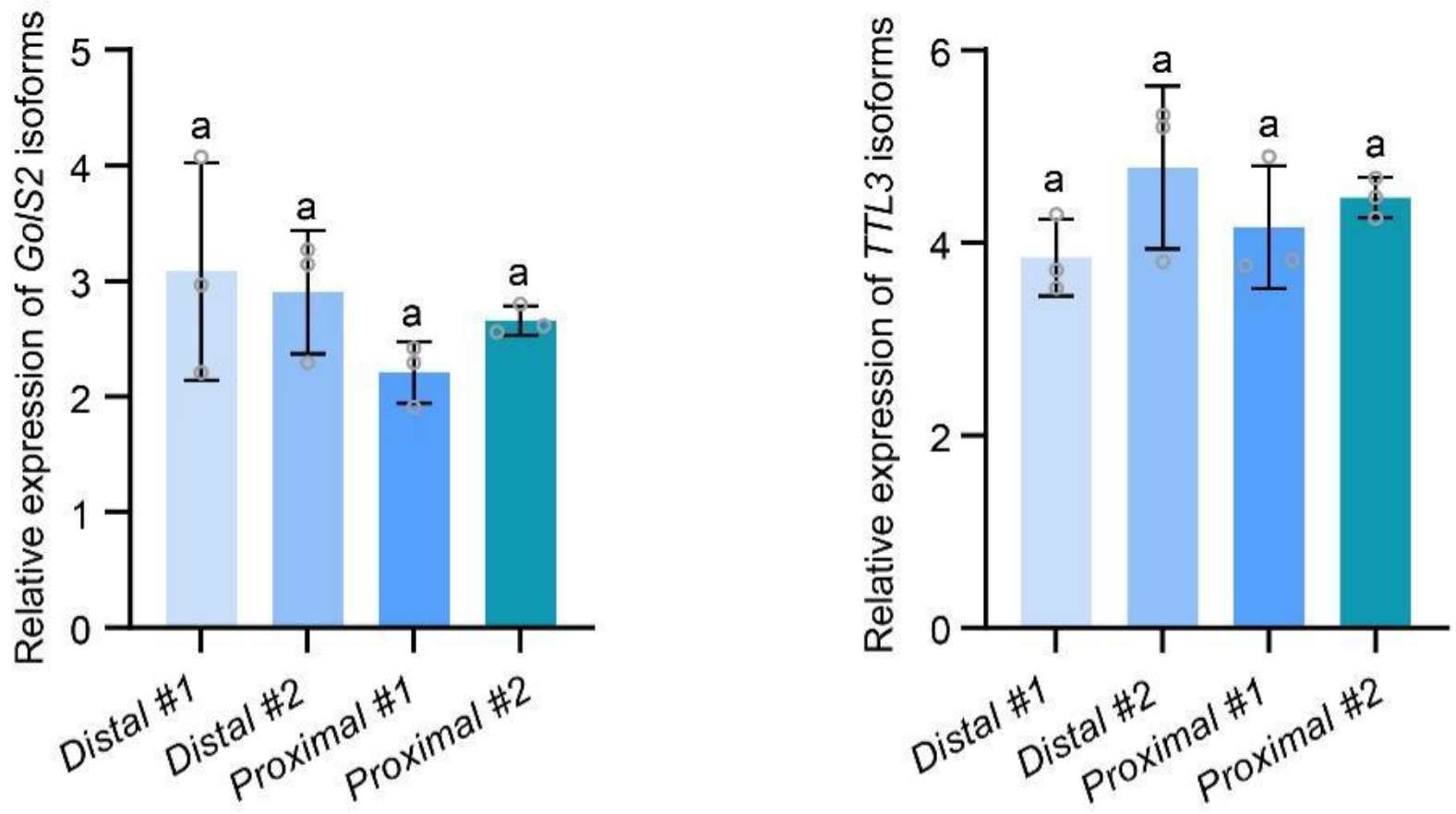

Fig. S9 Verification of transgenic plants overexpressing either the proximal or distal version of *GolS2* and *TTL3*. Transcript levels were quantified using RT-qPCR. The expression levels of *GolS2* and *TTL3* transcripts in WT were set as 1. *ACTIN2* served as the reference gene. Data are presented as mean ± SD from three technical replicates in a single experiment, with three biologically independent experiments yielding similar patterns. Significance was assessed using one-way ANOVA followed by Tukey's multiple comparisons test ($p < 0.05$).

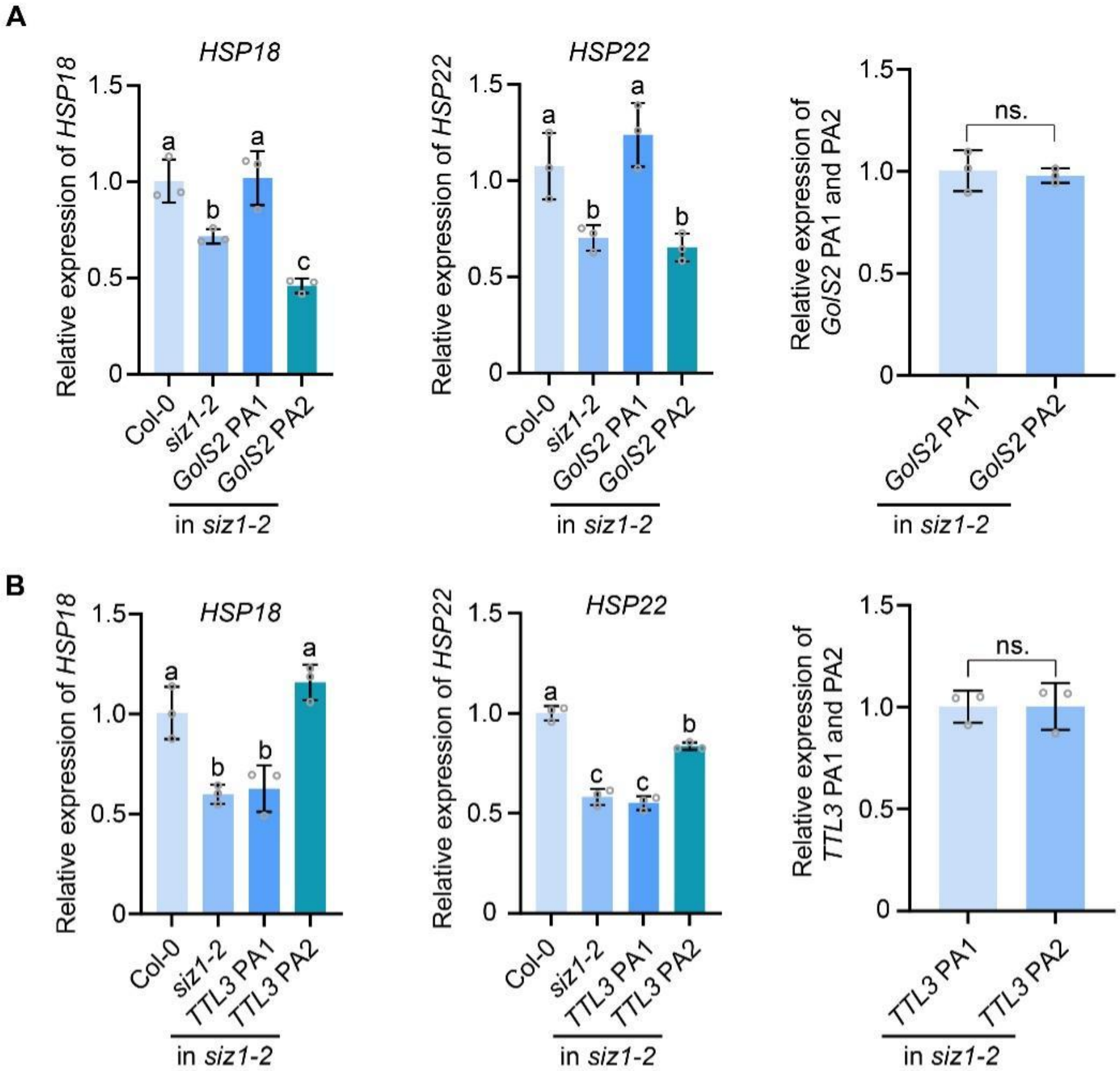

Fig. S10 The function of different transcripts of *GolS2* and *TTL3* in *siz1-2* mutant under HS. **A-B** Different isoforms of *TTL3* or *GolS2* were overexpressed in *siz1-2* cells cultured overnight at room temperature and then subjected to HS treatment (37°C) for 30 minutes. The relative expression of *HSP18* and *HSP22* is shown in the left and middle graphs, respectively. The relative expression levels of the overexpressed proximal (PA2) and distal (PA1) isoforms of *TTL3* or *GolS2* in *siz1-2* cells are shown in the right graph. *ACTIN2* served as an internal control. Data are mean ± SD from three technological replicates in a single experiment. Three biologically independent experiments yielded similar patterns. Significant differences were analyzed using one-way ANOVA followed by Tukey's multiple comparisons test ($p < 0.05$), and Student's *t*-test (ns., $p > 0.05$).

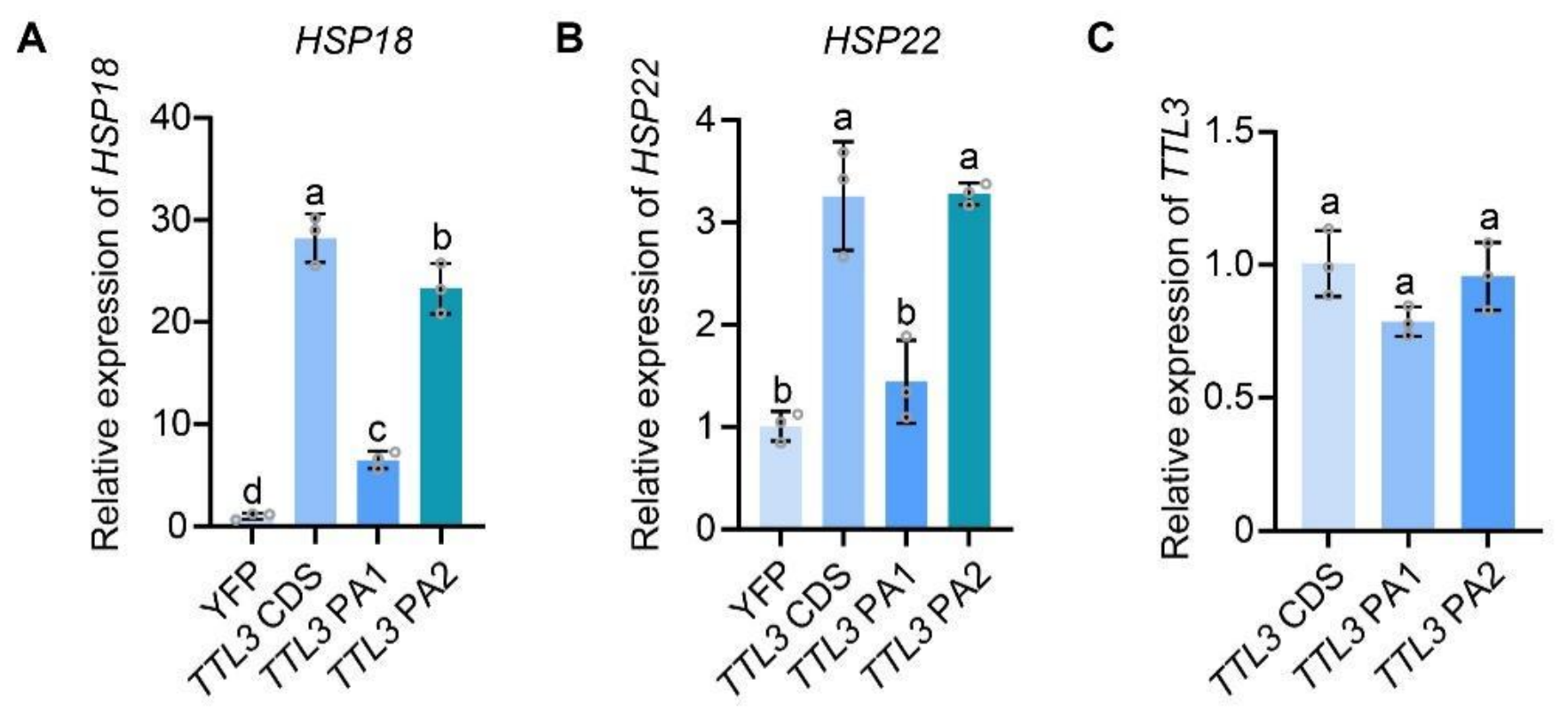

Fig. S11 Role of APA in regulating *TTL3* gene function. The PA1, PA2 and CDS of *TTL3* were each constructed into a YFP expression vector, and expressed in wild-type cells. The cells were cultured overnight at 22°C and subsequently subjected to HS treatment for 30 minutes. The YFP vector was used as a control. **A-B** The relative expression of *HSP18* and *HSP22* in wild-type cells overexpressing indicated *TTL3* isoforms expressing is shown in the left and middle graphs, respectively. **C** The relative expression levels of overexpressed *TTL3* isoforms in wild-type cells are shown in the right graph. The expression levels of downstream genes in the YFP vector were set to 1. Data are mean ± SD from three technological replicates in a single experiment. Three biologically independent experiments yielded similar patterns. Significant differences were analyzed using one-way ANOVA followed by Tukey's multiple comparisons test ($p < 0.05$).

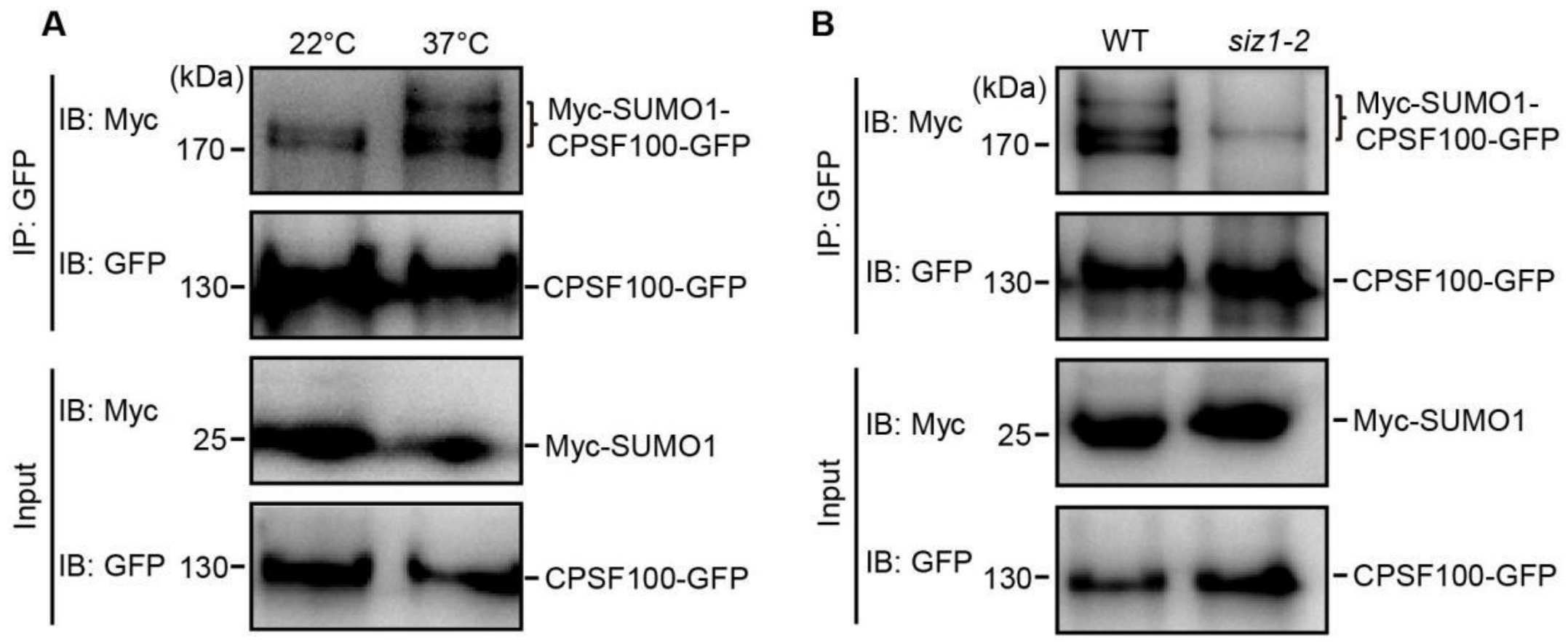

Fig. S12 Determination of CPSF100 SUMOylation in plant cells. **A** CPSF100-GFP was co-expressed with 5×Myc-SUMO1 in the wild-type cells overnight at 22°C as a control sample. For HS treatment, samples were first incubated overnight at 22°C and then were subjected to 37ºC for 30 min. **B** CPSF100-GFP was co-expressed with 5×Myc-SUMO1 in the wild-type or *siz1-2* cells over night at 22°C and subsequently subjected to 37°C treatment for 30 min. For SUMOylation detection, CPSF100-GFP was precipitated using anti-GFP beads. The input and pull-down samples were analyzed by immunoblots using anti-GFP and anti-Myc antibodies. The representative immunoblot images are shown.
